# Diversity Patterns of Dung Beetles along a Mediterranean Elevational Gradient

**DOI:** 10.3390/insects12090781

**Published:** 2021-08-31

**Authors:** Cristina Mantoni, Noelline Tsafack, Ettore Palusci, Stefano Di Pietro, Simone Fattorini

**Affiliations:** 1 Department of Life, Health and Environmental Sciences, University of L’Aquila, Via Vetoio, 67100 L’Aquila, Italy; cristina.mantoni@graduate.univaq.it (C.M.); ettorepalusci@gmail.com (E.P.); dipistefano@gmail.com (S.D.P.); 2 cE3c-Centre for Ecology, Evolution and Environmental Changes, Azorean Biodiversity Group, Faculdade de Ciências e Engenharia do Ambiente, Universidade dos Açores, rua Capitão João D’Avila, 9700-042 Angra do Heroísmo, Portugal; noellinetsafack@gmail.com

**Keywords:** Coleoptera, hill numbers, Scarabaeoidea, Geotrupidae, Scarabaeidae, Aphodiinae, altitude, Apennines, coprophagous beetles, biodiversity

## Abstract

**Simple Summary:**

Mountains are ideal natural laboratories to study how biodiversity is influenced by environmental characteristics because climate varies rapidly from lowlands to high elevations. Scientists have investigated how the number of species varies with elevation for the most disparate plant and animal groups worldwide. However, species richness is only one aspect of biodiversity that does not consider species abundances. The so-called Hill numbers are a unified family of mathematical indices that express biodiversity in terms of both richness and abundance. We used Hill numbers to investigate how dung beetle diversity varies along an elevational gradient in a Mediterranean mountain. We found that scarabaeids were the most abundant dung beetle group. These insects construct subterranean nests protecting their offspring from desiccation during warm and dry summer climatic conditions. Additionally, in accordance with their preference for open habitats, we found that dung beetles are more abundant and diversified in grasslands than in woodlands. In the woodlands, diversity increased with elevation because of tree thinning, whereas in the grasslands, diversity decreased because of increasingly harsher environmental conditions. This indicates a trade-off in the beetle response to elevation between the positive effects of increasing the availability of suitable habitats and the worsening of environmental conditions.

**Abstract:**

Most studies of biodiversity–elevational patterns do not take species abundance into consideration. Hill numbers are a unified family of indices that use species abundance and allow a complete characterization of species assemblages through diversity profiles. Studies on dung beetle responses to elevation were essentially based on species richness and produced inconsistent results because of the non-distinction between different habitats and the use of gradients dispersed over wide areas. We analyzed dung beetle diversity in a Mediterranean mountain (central Italy) for different habitats (woodlands vs. grasslands) and taxonomic groups (scarabaeids and aphodiids). Scarabaeids were the most abundant. Since scarabaeids are able to construct subterranean nests, this indicates that the warm and dry summer climatic conditions of high elevations favor species capable of protecting their larvae from desiccation. Dung beetles were more abundant and diversified in grasslands than in woodlands, which is consistent with their preference for open habitats. In the woodlands, diversity increased with increasing elevation because of increasing tree thinning, whereas, in the grasslands, diversity decreased with elevation because of increasingly harsher environmental conditions. These results indicate a trade-off in the beetle response to elevation between the positive effects of increasing the availability of more suitable habitats and the decrease of optimal environmental conditions.

## 1. Introduction

Patterns of variation in biodiversity along elevational gradients have long attracted the interest of naturalists, and they have become increasingly popular in recent years [1,2]. Alpha-diversity (i.e., the diversity of species within a particular area or ecosystem), beta-diversity (i.e., species turnover), functional diversity, animal body size, biogeographical composition, and several other aspects of community structure vary markedly with altitude in response to the associated variation in environmental parameters [3,4,5,6,7,8,9,10,11,12,13,14,15,16,17]. For example, climatic conditions change more rapidly with altitude than with latitude (the world average is a drop of about 1 °C for every 150 m above sea level against 1 °C for every 150 km poleward), which makes mountain areas ideal natural laboratories to investigate patterns of variation in biodiversity in response to environmental factors within geographically restricted areas [6,18].

Most of the studies investigating elevational patterns in biodiversity have dealt with species richness (i.e., number of species found at different altitudes) as a measure of alpha-diversity, finding either a monotonic decrease in species richness with increasing elevation or a hump-shaped trend with a mid-elevational peak [13,17,19]. The monotonic decrease is generally interpreted as a consequence of increasing harsher conditions (such as lower temperatures, higher radiation, low partial pressures of both oxygen and carbon dioxide, stronger winds, lower soil nutrients, less stable substrates, and shorter plant growing seasons), decreasing productivity, decreasing available area (because of the conical shape of mountains), and nested species distributions, in which higher assemblages tend to be progressively smaller subsamples comprising the most tolerant species among those already present in the lower ones [13,18]. By contrast, a hump-shaped pattern is frequently interpreted as a result of purely stochastic processes (species ranges tend to overlap in domain centers due to dispersal limitations, resulting in a mid-domain richness peak) [13,18].

Species richness is, however, a problematic measure of the diversity of an assemblage because it depends on sampling intensity and does not take species abundance distribution into consideration [20]. The first problem (i.e., the effect of sampling effort) can be addressed by using rarefaction/extrapolation procedures to obtain richness values that are standardized to a common sampling effort or by calculating asymptotic estimators that are relatively independent of additional sampling efforts [20]. The second problem (i.e., the abundance problem) has led many ecologists to propose measures (indexes) of diversity that combine species richness and the proportion of each species into a single metric [21,22].

Among the vast multitude of diversity measures proposed to take the abundance of the species in an assemblage into account, Hill numbers *^q^D* are a mathematically unified family of indices that (1) combine information on species richness, species rarity, and species dominance and (2) are all expressed in the same units (i.e., effective number of species); therefore, they are comparable between each other [20,23]. As the diversity order *q* can assume any value, it is possible to obtain a complete characterization of an assemblage by constructing a diversity profile plotting *^q^D* versus *q*. For these reasons, Hill numbers are increasingly used in community ecology research [20,24].

In this paper, we used Hill numbers to investigate the elevational patterns of diversity of dung beetles in a Mediterranean montane area. Dung beetles are one of the most popular beetle groups among insect ecologists [25] because of their taxonomic richness (with 7000 known species worldwide [25]), the many essential ecosystems services they provide (such as nutritional cycling [26,27], their soil maintenance characteristics [28,29,30,31,32,33,34], seed dispersal [35], and their ability to protect livestock from dipterans that develop in dung [36]), their responses to environmental changes [37,38,39], and their sensitivity to anthropogenic disturbances [40,41,42,43]. Therefore, it is not surprising that dung beetle assemblages have been the object of various research studies dealing with elevational patterns of diversity.

Most studies on dung beetle diversity in the mountainous areas in Europe, North America, Southeast Asia, South Africa, and South America have highlighted a decrease in the number of species with increasing altitude (e.g., [37,44,45,46,47,48,49,50,51,52,53,54]). In the Western Palearctic, dung beetle richness was found to decline with increasing elevation in the Northern French Alps [44,45], in the Southern French Alps [46], in the Sierra Nevada (Spain) [46,47], in Mediterranean grassland sites in the Southern French Alps [49], and in the Kütahya Province in western Turkey [53]. Hump-shaped patterns were, however, found in the northern French Alps (with the mode within 1500–1800 m along a gradient from about 700 to about 3000 m) [46,48], in the Western Rhodopes Mountains (with the mode at 900 m along a gradient from 200 to 2000 m) [55], and in the Iberian Central System (with the mode at 1000 m along a gradient from 500 to 2200) [55]. No effect of altitude on species richness was observed in a mountainous area near the Cantabrian Sea (namely the western portion of the Picos de Europa, Spain) [56] and in Sierra de Baza (south-eastern Iberian Peninsula) [57].

These studies, however, were characterized by various sources of complexity that make comparisons and interpretations difficult. First, with the exception of a single study [56], all of the available research (e.g., [44,45,53,55,57,58]) used elevational gradients extended over wide areas, sometimes including regions with different ecological settings and biogeographical histories. When gradients are constructed by taking localities dispersed over wide areas, the communities sampled at different elevations might belong to faunas influenced by different biogeographical and evolutionary processes [58].

Second, many studies on the elevational patterns of dung beetle diversity did not consider the differences in the type of habitat with which dung beetles are associated (e.g., [44,45,46,55]), while others investigated a single habitat [49] or compared the response between different habitats [53,56]. Most European dung beetles are generally associated with open habitats, and it has been reported that as vegetation cover increases, species richness and total abundance decrease [59,60,61,62]. For example, Romero-Alcaraz and Ávila [57] found that Mediterranean dung beetle communities are characterized by higher values of abundance and diversity in open habitats (e.g., open scrublands with little vertical development and open pastures) than in closed (forest) habitats. Moreover, responses to habitat type may change with elevation [62]. Even the local climatic setting, which is highlighted by the presence of different vegetation types, influences diversity patterns. For example, in the mountain grasslands of the Southern Alps, Errouissi et al. [49] found that dung beetle diversity (expressed using the Margalef index, which balances the richness by the number of sampled individuals) decreased with increasing elevation for Mediterranean sites but increased in temperate sites. Thus, results from different studies may appear contradictory if they were conducted in different habitats, or are difficult to interpret if data from different habitat types were merged.

Third, available studies were mostly conducted using species richness as a measure of diversity, with only cursory notes on other aspects of diversity (e.g., negative influences of elevation on the Shannon index and the Shannon-based equitability [44,45] as well as on the Margalef index [49] for Alpine dung beetle assemblages; negative influence on the Shannon index in an Afromontane forest [54]; no influence of elevation on the Simpson index in a tropical area in Brazil [52]). To the best of our knowledge, no research used diversity profiles.

In this paper, we applied a diversity profile analysis based on Hill numbers to dung beetle assemblages sampled along an elevational gradient at a narrow geographical scale to overcome the problem of mixing communities belonging to different faunas. Additionally, we conducted separate analyses for open and closed habitats to assess if elevational patterns differed between habitat types.

Specifically, we tested the following two hypotheses:

(1) In open habitats (grasslands), dung beetle diversity should show a decline in biodiversity with increasing elevation as a consequence of increasing harsher conditions (lower temperatures and higher insulation);
(2) If most of dung beetles prefer open habitats and if the disadvantages of harsher climatic conditions are overwhelmed by the advantages provided by a more favorable habitat type, dung beetle diversity in closed habitats (forests) should show an opposite pattern (i.e., biodiversity should increase with increasing elevation) because tree cover decreases with elevation.

We tested these hypotheses by both comparing diversity curves at different elevations and by investigating elevational variations for specific Hill numbers corresponding to species richness, Shannon diversity, and Simpson diversity in closed and open habitats.

Because of the influence of the sample size in diversity estimates, we conducted our study by using (1) empirical estimates obtained by standardizing data on the basis of the sampling effort (number of active traps over the entire sampling period at each elevation); (2) estimates based on rarefaction/extrapolation to an equal coverage; and (3) asymptotic estimates.

## 2. Materials and Methods

### 2.1. Study Area

The study was conducted along an elevational gradient of about 1000 m (from about 950 m to 2000 m) in the Central Apennines (Latium, Central Italy) within the “Montagne della Duchessa” Regional Nature Reserve. The reserve has a surface of 35.4 km^2^ and an altitudinal range of 1450 m (from 950 to 2239). The Montagne della Duchessa mountains are a Cretaceous carbonatic massif with a landscape composed of peaks above 2000 m and high-sloped valleys created by glacial and karstic erosion.

Between 800 and 1200 m, the vegetation is mainly represented by mixed woods, dominated by oaks (*Quercus cerris*, *Quercus pubescens*, and *Quercus ilex*) associated with hornbeams (*Ostrya carpinifolia*), ash trees (*Fraxinus excelsior*), and maples (*Acer pseudoplatanus*, *A. campestris*, and *A. monspessulanum*). Open habitats are occupied by grasslands corresponding to the Habitat of Community Interest 6210, defined as ‘semi-natural dry grasslands and scrubland facies on calcareous substrates (*Festuco-Brometalia*)’, according to the European Commission classification [63,64]. From 1200 to 1500 m, woods are dominated by the hornbeam, and above 1500, the beech (*Fagus sylvatica*) is the dominant species. The tree line occurs at 1700–1800 m. Above the tree line, vegetation is represented by juniper brushlands and calcareous grasslands. Secondary meadows due to extensive pasture occur along all the gradient, but these are particularly common at the highest elevations.

The large herbivores present in the study area during the sampling period included about 300–400 deer, 80–100 roe deer, and 300 wild boars as sedentary species. Domesticated herbivores included about 400–450 sheep, 300 cows, and 150 horses, which are moved from lowland to high altitude grasslands in the summer months (June–September).

### 2.2. Sampling Protocol

The elevational gradient was divided into seven intervals (belts) of equal size (150 m, 950–1100 m, 1100–1250 m, 1250–1400 m, etc.). In each belt, dung beetles were sampled using two baited traps: one placed in a grassland and the other placed in a woodland, with the exception of the highest belt, which was above the tree line and therefore entirely occupied by grasslands.

Beetles were collected using ‘bait-surface-grid’ traps [57,65] made up of buckets (diameter: 186 mm, depth:168 mm) sunk in the ground with their rim at soil level. Each trap was filled with 400 mL of water to prevent dung beetles from escaping the trap after falling in. Traps were covered with a 20 mm mesh iron net, and 1 kg of fresh bovine dung was placed on the net. This type of trap is considered to be one of the most efficient methods to sample dung beetles [57,65,66].

Sampling was conducted once a month from June to November 2018. Each trap was active for 48 h; after this time, the traps were emptied to collect the beetles that had fallen into the buckets, which were left on site to be re-used in the following month [52,57,67,68]. The location of the traps therefore remained constant over the entire sampling period. The samples were washed and stored in 70% ethanol. The dung beetles were identified to the species level using keys [69]. All material is preserved in the senior author’s (SF) collection.

### 2.3. Data Analysis

We performed analyses both by maintaining forest and grassland samples separately and by merging the two habitats to investigate the impact of not distinguishing habitat types in the study of dung beetle assemblages. In all cases, temporal replicates were aggregated prior analyses. To take into account the loss of certain traps in some months and the lack of wooded habitats in the highest belt, for each belt, the number of collected individuals in a given habitat was standardized proportionally to the minimum number of active traps in that habitat. The same procedure was adopted for analyses in which data from the different habitats were merged.

To analyze how dung beetle diversity varied along the elevational gradient, we calculated diversity profiles using Hill numbers (*^q^D*) for each dung beetle sample. Hill numbers differ among themselves only by an exponent, the diversity order *q*, which determines the measure’s sensitivity to the relative abundances of species. To obtain a complete characterization of an assemblage, a diversity profile is constructed by plotting *^q^D* versus *q* from *q* = 0 to *q* = 3 or 4 (beyond this it changes very little, whereas *q* < 0 are not used, as they are dominated by the abundances of rare species and have poor statistical sampling properties) [24]. We constructed diversity profile curves for *q* varying between 0 and 3 with increments of 0.25.

A first set of analyses was conducted by calculating the Hill numbers on the data standardized as described above (empirical estimates). As Hill numbers are influenced by sample size, we performed a second set of analyses using a rarefaction/extrapolation procedure [20] on raw data. Extrapolation should not exceed doubling [20]; thus, we used the double of the minimum value of sampled individuals as a common value for rarefaction/extrapolation. Finally, we developed a third set of analyses using asymptotic values [20] to deal with the possibility of undersampling. For each index, the associated 95% confidence intervals were also calculated [20]. Analyses were conducted using the R packages SpadeR version 0.1.1 [70] with the function *Diversity* to compute empirical and asymptotic diversity estimates and iNEXT version 2.0.20 [20,71] with the function *iNEXT* to compute rarefied and extrapolated diversity estimates. The R package ggplot2 version 3.3.3 [72] was used for the graphics.

Hill numbers include three widely used species diversity measures as special cases: species richness (*q* = 0), exponential Shannon diversity (*q* = 1 as the limit case since the equation for Hill numbers is undefined for *q* = 1), and Simpson diversity (*q* = 2) [20,23]. Thus, we modelled how these three particular measures varied with elevation by using generalized linear models (GLMs). We used a Poisson distribution and a log-link for species richness (rounded to integers for estimates obtained after rarefaction/extrapolation or as asymptotes), whereas a Gaussian distribution was used for the Shannon and Simpson diversity. The last procedure was also used to model how standardized abundances varied with elevation. GLMs were applied by using the R package stats [73], and model performances were assessed using a Kullback–Leibler-divergence-based *R*^2^. *R*^2^ estimates were computed using the R package rsq version 2.2 with the function *rsq* [74].

Analyses were conducted for all dung beetles (geotrupids + aphodiids + scarabaeids) and for aphodiids and scarabaeids separately. Systematics of Scarabaeoidea is much discussed; more traditional authors include all of the main groups as subfamilies of a few families (with the whole Aphodiidae considered as a subfamily—Aphodiinae—of Scarabaeinae), while others tend to identify a higher number of families (with Aphodiidae as a family on their own hence the separation from the Scarabaeidae) [75]. We adopted this second approach, which is the most commonly used by European taxonomists [75] and that was previously followed in various ecological studies (e.g., [45,46,49,56,57]). However, in our case, the choice is merely nomenclatorial and does not affect the results.

## 3. Results

In total, we collected 12,324 dung beetles belonging to 42 species (Table 1; Table S1). Aphodiids represented 66% of the collected species and 18% of the collected individuals; scarabaeids accounted for 27% of the collected species and 81% of the collected individuals; geotrupids were 7% of the collected species and less than 2% of the collected individuals. Thus, geotrupids were the less represented taxon in terms of both richness and abundance. Aphodiids were more diversified, but they were less abundant than scarabaeids. The proportional abundance of aphodiids was lower than that of scarabaeids at all elevations except for the first belt, where aphodiids accounted for about 67% of total dung beetles, whereas scarabaeids were 25%. The proportional abundance of aphodiids tended to decline with elevation, whereas that of scarabaeids tended to increase, although the relationships were marginally not significative (Spearman rank correlation tests: Aphodiidae *r*_s_ = −0.750, Scarabaeidae *r*_s_ = 0.750; *p* = 0.052 in both cases). Dung beetles were much more abundant in grasslands than in woodlands (average values of standardized abundances for belts 1–6 ± standard error: 1483.972 ± 636.049 in grasslands, 112.639 ± 24.667 in woodlands; Wilcoxon test for paired data: *p* = 0.028).

**Figure 1:**
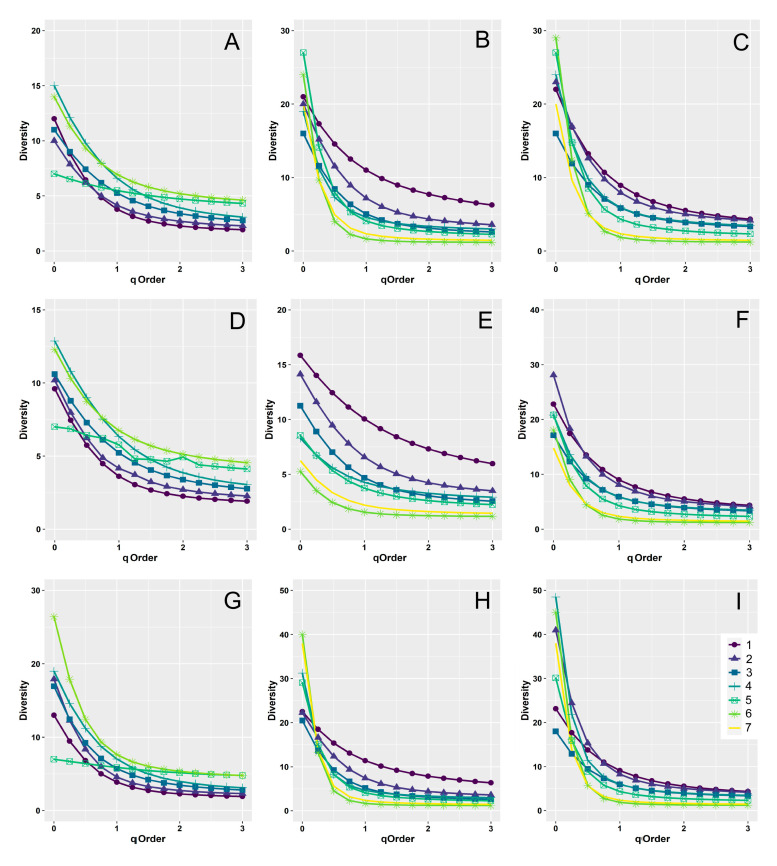
Diversity curves using Hill numbers for dung beetle assemblages along an elevational gradient in central Italy. Curves were constructed using empirical (**A**–**C**), rarefied/extrapolated (**D**–**F**), and asymptotic (**G**–**I**) values for the two habitats (woodlands and grasslands) separately (woodlands: **A**,**D**,**G**; grasslands: **B**,**E**,**H**) and merged (**C**,**F**,**I**). Dung beetles were sampled in seven altitudinal intervals (belts) of 150 m extent: Belt 1: 950–1100 m, Belt 2: 1100–1250 m, Belt 3: 1250–1400 m, Belt 4: 1400–1550, Belt 5: 1550–1700, Belt 6: 1700–1850, Belt 7: 1850–2000. For ease of reading, 95% confidence intervals are not shown here, but they are reported in Figure S1.

Diversity curves constructed with different approaches (empirical, rarefied/extrapolated, and asymptotic values) showed similar patterns, with substantial differences only occurring for *q* = 0, which clearly indicates the impact of sampling size on species richness estimates (Figure 1).

Using empirical values, woodland diversity curves clearly indicated a direct relationship between diversity and elevation (Figure 1A) for *q* ≥ 1.5; however, the confidence intervals were largely overlapping (Figure S1A). By contrast, grassland diversity curves (Figure 1B) showed distinct patterns of decreasing diversity with increasing elevation. For *q* = 0, diversity values were characterized by large uncertainties (Figure S1B) and did not show any clear relationship with elevation. However, with increasing *q*-values, confidence intervals became narrower, and diversity values showed distinct elevational patterns. The first belt (950–1100 m) showed the highest diversity followed by the second belt (1100–1250 m). Belts 3–5 (1250–1400, 1400–1550, 1550–1700 m, respectively) showed intermediate values with largely overlapping confidence intervals. The seventh belt (1850–2000 m) showed a lower diversity, and the sixth belt (1700–1850 m) was that with the lowest diversity. When the two habitats were grouped, the emerging pattern was similar to that obtained for the grasslands (Figure 1C and Figure S1C). The first two belts (950–1100 m and 1100–1250 m, respectively) showed overlapping confidence intervals, both with clearly higher diversity values at increasing *q*-values. The third and fourth belts (1250–1400 and 1400–1550 m, respectively) presented largely overlapping confidence intervals, with diversity values only slightly lower than those of the first and second belts. The fifth belt (1550–1700 m) had a substantially lower diversity, and the sixth and seventh belts (1700–1850 and 1850–2000 m, respectively) showed the lowest diversity.

The use of a rarefaction/extrapolation approach (Figure 1D–F and Figure S1D–F) produced similar results, but with clearer patterns. Again, woodland diversity curves (Figure 1D and Figure S1D) indicated a clear positive relationship between diversity and elevation for *q* ≥ 1.5 although confidence intervals overlapped by a large amount. By contrast, in the grassland habitat (Figure 1E and Figure S1E), diversity tended to decrease with elevation, even for *q* = 0. When the two habitats were grouped (Figure 1F and Figure S1F), the emerging pattern was similar to that obtained for the grasslands but with the first and second belts showing more similar diversity values. Curves of asymptotic values (Figure 1G–I, Figure S1G–I) differed from the previous ones, especially for the exaggerated estimates of diversity at *q* = 0, with extremely large confidence intervals (Figure 1G and Figure S1G).

**Figure 2:**
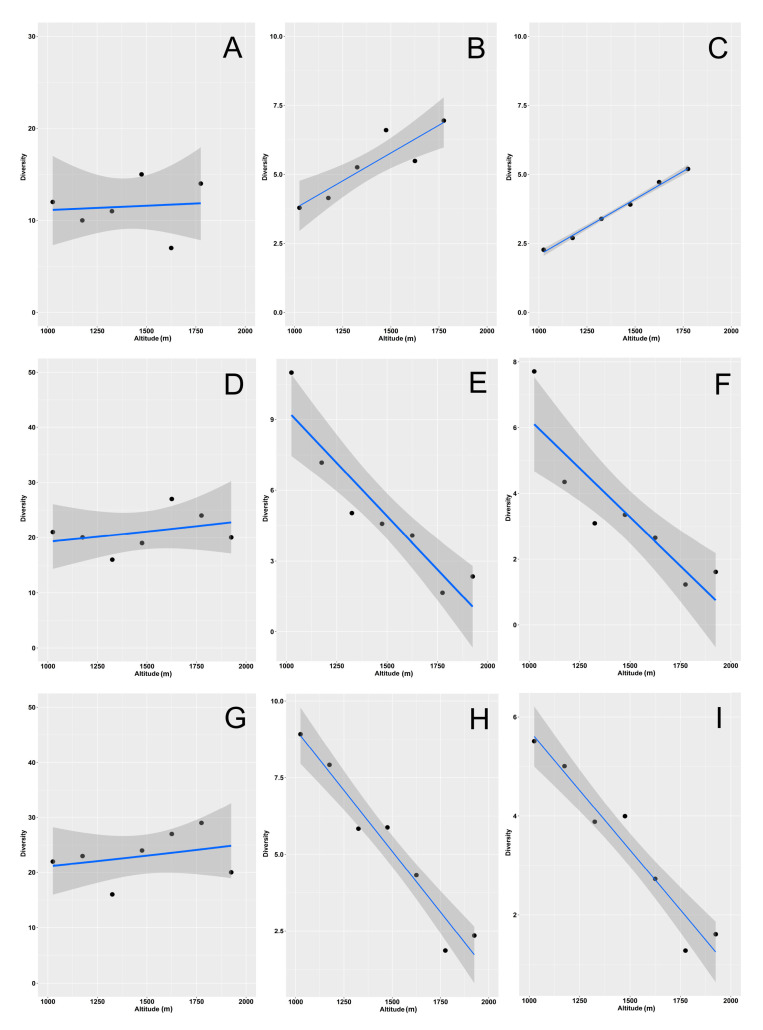
Relationship of dung beetle species richness (**A**,**D**,**G**), exponential Shannon diversity (**B**,**E**,**H**), and Simpson diversity (**C**,**F**,**I**) with altitude along an elevational gradient in central Italy. Curves were constructed using empirical values for the two habitats (woodlands and grasslands) separately (woodlands: **A**–**C**; grasslands: **D**–**F**) and merged (**G**–**I**). General linear models were used for fitting. Dashed areas are 95% confidence intervals.

We focused on the diversity values obtained for *q* = 0 (species richness), *q* = 1 (exponential Shannon diversity), and *q* = 2 (Simpson diversity) to model how they varied with elevation (Figure 2, Figures S2 and S3, Table 2).

In the woodlands, species richness did not show any elevational trend (Figure 2A, Figures S2A and S3A), whereas both the Shannon (Figure 2B, Figures S2B and S3B) and Simpson (Figure 2C, Figures S2C and S3C) diversity increased with elevation (see also Table 2). By contrast, in the grasslands, all three indices decreased with elevation when using values obtained with rarefaction/extrapolation (Figure S2D–F, Table 2).

**Figure 3:**
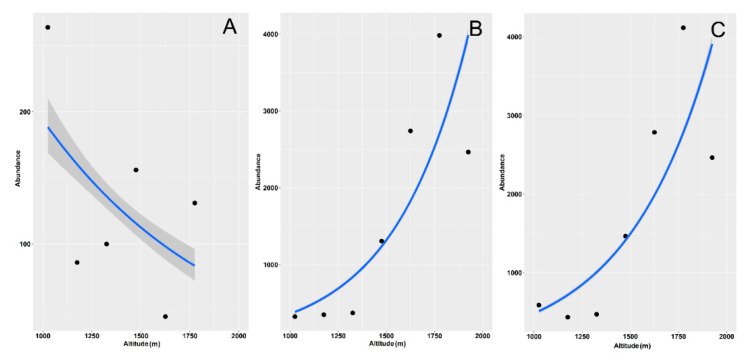
Relationship between dung beetle species abundance and altitude along an elevational gradient in central Italy. Curves were constructed using standardized abundance values for the two habitats (woodlands and grasslands) separately (woodlands: (**A**), grasslands: (**B**) and merged (**C**)). General linear models were used for fitting. Dashed areas are 95% confidence intervals. Confidence intervals in panels (**B**,**C**) are not distinguishable because of their narrowness.

Using empirical (Figure 2E,F) and asymptotic (Figure S3E,F) values, the Shannon and Simpson indexes had the same behavior, whereas richness exhibited no significant relationship with elevation using empirical estimates (Figure 2D) and a positive relationship using asymptotic values (Figure S3D) (see also Table 2). When the two habitats were merged, both the Shannon and Simpson diversity decreased with elevation (Figure 2H,I, Figures S2H,I and S3H,I), whereas richness showed a significant decrease only when calculated using rarefaction/extrapolation (Figure S2G) (see also Table 2). Abundance increased significantly with altitude for grasslands and for the two habitats merged, but it did not increase for the woodlands (Figure 3, Table 2).

**Figure 4:**
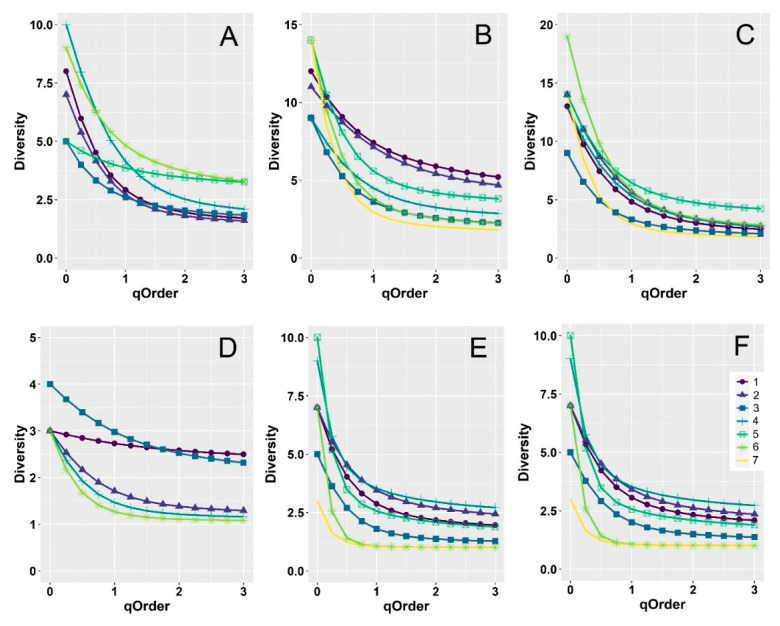
Diversity curves using Hill numbers for dung beetle assemblages along an elevational gradient in central Italy. Curves were constructed for aphodiids (**A**–**C**) and scarabaeids (**D**–**F**) using empirical values for the two habitats (woodlands and grasslands) separately (woodlands: (**A**,**D**); grasslands: (**B**,**E**) and merged (**C**,**F**)). Dung beetles were sampled in seven altitudinal intervals (belts) of 150 m extent: Belt 1: 950–1100 m, Belt 2: 1100–1250 m, Belt 3: 1250–1400 m, Belt 4: 1400–1550, Belt 5: 1550–1700, Belt 6: 1700–1850, Belt 7: 1850–2000. For ease of reading, 95% confidence intervals are not shown here, but they are reported in Figure S4.

When the aphodiids and scarabaeids were analyzed separately, diversity profiles did not show any clear pattern, with largely overlapping confidence intervals occurring (Figure 4 and Figures S4–S8). Only the curves for the 3rd, 6th, and 7 h belts in the aphodiids (for grasslands and the two habitats merged) appeared well separated (lower diversity) from the others.

**Figure 5:**
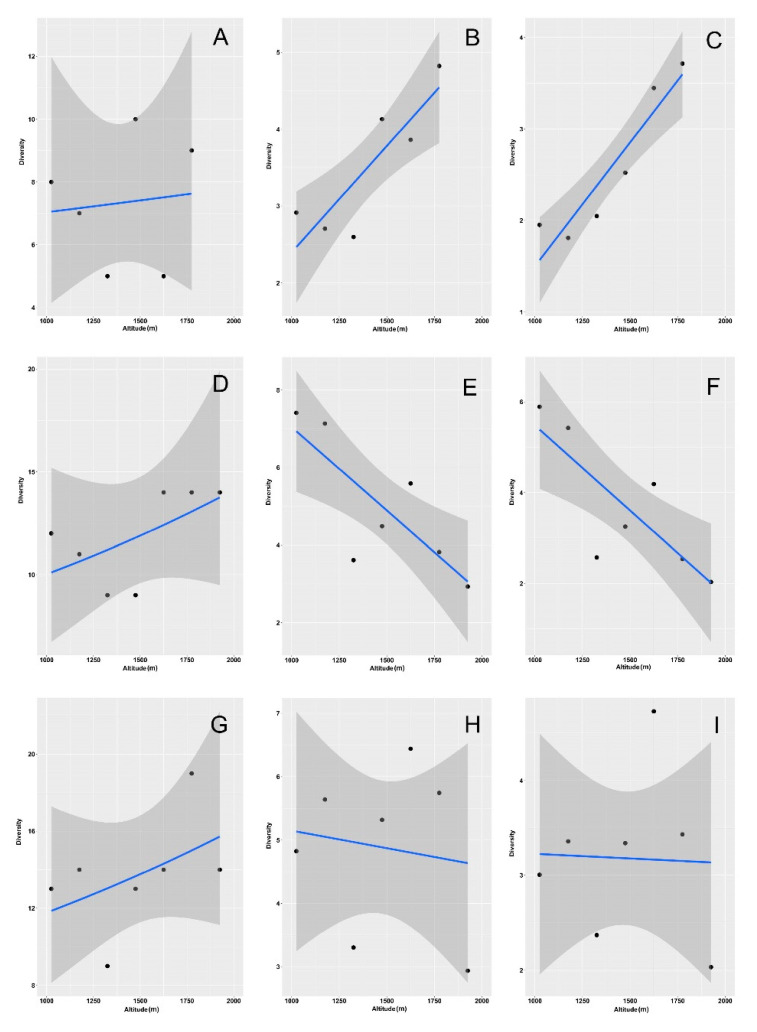
Relationships between aphodiid species richness (**A**,**D**,**G**), exponential Shannon diversity (**B**,**E**,**H**), and Simpson diversity (**C**,**F**,**I**) and altitude along an elevational gradient in central Italy. Curves were constructed using empirical values for the two habitats (woodlands and grasslands) separately (woodlands: **A**–**C**; grasslands: **D**–**F**) and merged (**G**–**I**). General linear models were used for fitting. Dashed areas are 95% confidence intervals.

**Figure 6:**
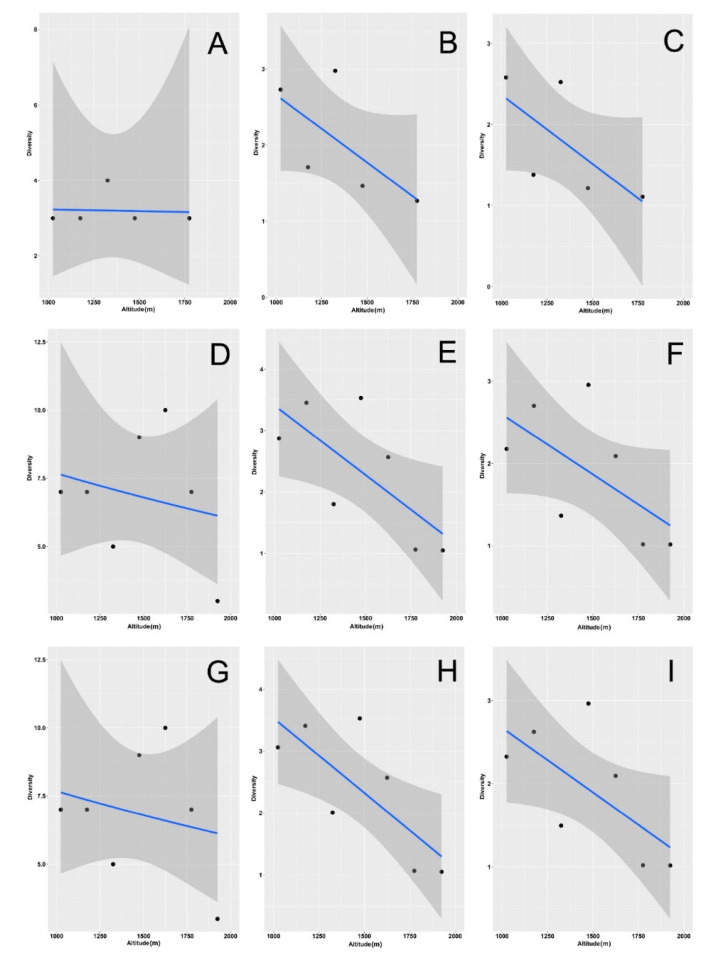
Relationship of scarabaeid species richness (**A**,**D**,**G**), exponential Shannon diversity (**B**,**E**,**H**), and Simpson diversity (**C**,**F**,**I**) and altitude along an elevational gradient in central Italy. Curves were constructed using empirical values for the two habitats (woodlands and grasslands) separately (woodlands: **A**–**C**; grasslands: **D**–**F**) and merged (**G**–**I**). General linear models were used for fitting. Dashed areas are 95% confidence intervals.

Relationships between aphodiid diversity and elevation (Figure 5, Figures S9 and S10, Table 3) followed the patterns obtained with all dung beetle species, but the decline in the Shannon and Simpson indices for the aggregated habitats were not significant because of the opposite trends found in the woodlands and in the grasslands. In the scarabaeids (Figure 6, Figures S11 and S12, Table 4), all of the trends were negative (except for richness in woodlands with empirical and asymptotic estimates) but not significant (except for Shannon estimates in aggregated habitats, which were marginally not significant with empirical values and marginally significant with asymptotic values).

**Figure 7:**
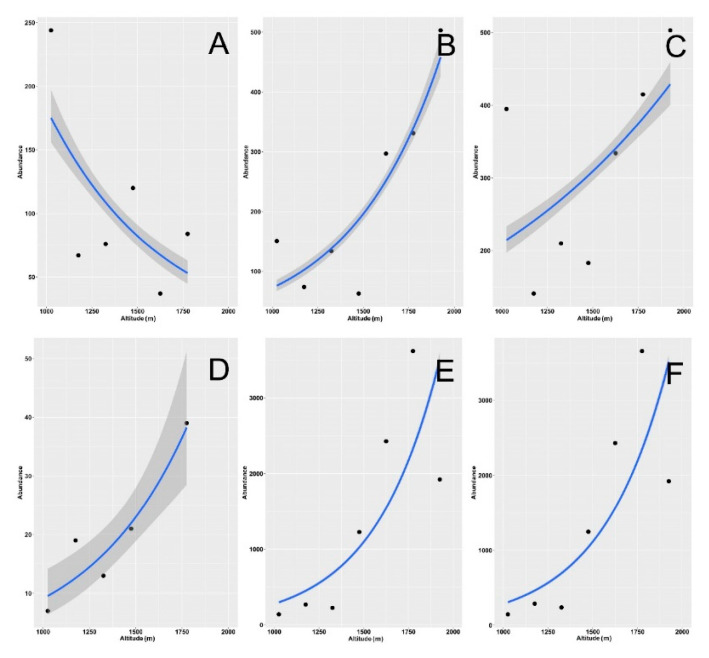
Relationship between dung beetle species abundance and altitude along an elevational gradient in central Italy. Curves were constructed separately for aphodiids (**A**–**C**) and scarabaeids (**D**–**F**) using standardized abundance values for the two habitats separately (woodlands: **A**,**D** and grasslands: **B**,**E**) and merged (**C**,**F**). General linear models were used for fitting. Dashed areas are 95% confidence intervals. Confidence intervals in panels E and F are not distinguishable because of their narrowness.

Aphodiid abundance declined with elevation in woodlands (Figure 7A, Table 3) but increased in grasslands (Figure 7B) and in the two habitats when merged (Figure 7C), whereas scarabaeids increased in both habitats and globally (Figure 7D–F, Table 4).

## 4. Discussion

The most diversified dung beetle group in the study area was the aphodiids, with 27 species, followed by the scarabaeids, with 12 species; the geotrupids were present with 3 species. This situation is representative of the whole Italian fauna, which includes 144 aphodiids, 52 scarabaeids, and 19 geotrupids [69]. Although less diversified, the scarabaeids were much more abundant than the aphodiids. Dung beetle communities in northern Europe are almost completely dominated by aphodiids, whereas scarabaeids dominate in tropical and Mediterranean ecosystems [76]. This pattern can be related to differences in excrement utilization between aphodiids and scarabaeids. According to excrement utilization, dung beetles can be roughly classified into three main categories: dwellers, tunnellers, and rollers [25,76,77], but this classification may be imprecise [78]. Scarabaeids are typically tunnellers (i.e., they dig chambers more or less directly underneath the pat for feeding or breeding) or rollers (i.e., they form a ball of dung that can be rolled away from the pat and buried for feeding or breeding), whereas most aphodiids are dwellers (i.e., they feed in the dung pat as adults and lay eggs within or under the dung mass where they undergo larval development and do not construct nests) [25,46,76,77], but there are aphodiids that construct more or less elaborate pedotrophic nests [78]. In our samples, the only aphodiid species that could not be classified as dwellers were *Colobopterus erraticus*, *Acrossus rufipes*, and *Bodilopsis rufa* [78].

In a cold and humid climate, excrement remains humid for long periods, dung removal is absent or slow, and thus competition among dung beetles is considered reduced; by contrast, in tropical and Mediterranean areas, excrement tends to dry and to be removed quickly, which possibly intensifies competition, favoring species able to protect the offspring through subterranean pedotrophic nests [76,79,80]. This may explain why temperate communities are dominated by dwellers (aphodiids), whereas tropical and Mediterranean communities are dominated by rollers and tunnellers (scarabaeids). The higher abundance of scarabaeids in our study area is consistent with this general pattern and is in line with previous findings in other Mediterranean mountain areas [58].

In Mediterranean areas, climate tends to be more temperate at high altitudes; thus, one might expect a predominance of aphodiids at the highest elevations because of the presence of colder climates. For example, Martin-Piera et al. [57] found that high elevation communities in the Iberian mountains were dominated by aphodiids and geotrupids, and Barbero et al. [81] found that aphodiids were numerically dominant in an Alpine valley (Sagone Valley, Western Alps). In our study, we found that aphodiids were only more abundant than scarabaeids at the lowest elevation, possibly because of the presence of more humid conditions allowed by the tree cover. The higher abundance of scarabaeids at the highest elevations suggests that conditions remain less favorable to aphodiids even with increasing elevation. This can be explained by the fact that average lower temperatures at higher elevations do not prevent excrement from a rapid desiccation, which is probably because of the drying effects of increased windiness and insulation. Thus, even high-altitude Mediterranean dung beetles must cope with the rapid desiccation of the excrement, especially in the summer, when precipitation may be completely absent. In these hot, dry, and insulated environments, scarabaeids can be favored because the use of subterranean pedotrophic nests may reduce the risk of larval death by desiccation.

On the whole, dung beetles were much more abundant in grasslands than in woodlands, which is consistent with the overall preferences of these beetles for open habitats [57,62] and the less abundant presence of potential food in the woodlands. Although deer and roe deer were relatively common in forests, sheep, cows, and horses, which were frequently observed in large numbers in open areas through the entire elevational gradient, were much less frequently observed in the forests, where their pats were also much less abundant. Because of the higher dung beetle abundance in grasslands, the results obtained with the two habitats merged mirrored those obtained for the grasslands alone, but they were less clear because of the confounding effects of the opposite patterns found in the woodlands. The physical structure of habitats can be an important determining factor in the composition and distribution of dung beetle assemblages [49,54,56,57]. Our results highlight the importance of distinguishing open assemblages from the forest ones.

In most cases, previous studies on the altitudinal distribution of dung beetle communities showed a decrease in species with increasing elevation (e.g., [48,50,51,53]), but mid-elevation peaks have been also documented in two Mediterranean areas [55], and in some cases, no relationship was found [57]. In our study, species richness decreased distinctly with elevation in the grassland habitat, whereas no clear pattern was found for the woodland assemblages. Mid-elevation peaks recorded in Mediterranean dung beetles were observed at around 800–900 m in the Iberian Central System and less clearly at around 900–1100 in the Western Rhodopes; above these altitudes, dung beetle richness decreased monotonically [55]. The absence of a mid-elevation peak in our study can be attributed to the fact that our elevational gradient started from 950 m, which is beyond these mid elevations. The difference in the species–elevation patterns observed in grasslands (decreasing number of species with increasing elevation) and woodlands (no relationship) are consistent with trends observed in a Western Anatolian mountain area, where species richness decreased with elevation in open habitats but not in forest habitats [53].

Woodland and grassland assemblages showed different diversity profiles. In general, the diversity curves of woodland assemblages were below those of grassland assemblages. A lower diversity of dung beetle assemblages in wooded habitats seems to be a common situation in the Western Palearctic region, where most of the dung beetles prefer open habitats [57,62], and the presence of open habitats have been increasing by millenary activities of grazing and deforestation [82]. Additionally, the woodland and grassland assemblages showed opposite responses to increasing elevation. In the woodlands, diversity tended to increase with elevation, while in the grasslands, diversity tended to decrease with elevation. Although these opposite patterns can be observed along almost all *q*-value ranges, they become clearer at increasing *q*-values, especially for *q* ≥ 1, which indicates the importance of the contribution of the most dominant species in generating the elevational increase (woodlands) or decrease (grasslands) in diversity. Examination of the elevational variation in the Shannon (*q* = 1) and Simpson (*q* = 2) diversity showed an increase in diversity for the woodland assemblages and a decrease for the grassland assemblages. These contrasting patterns can be interpreted as a result of a trade-off in the dung beetle response to elevation between the positive influence of the increasing availability of open habitats, which become more frequent with increasing elevations (although the tree-line in the study area is at around 1700–1800 m, woodland cover tends to decrease with elevation along the whole gradient), and the decrease of optimal environmental conditions (because of higher temperature, lower water availability, and higher insulation). A recent study on Afromontane dung beetle assemblages, where dung beetle abundance, richness, and Shannon diversity declined with elevation, showed positive correlations between mean ground cover and dung beetle abundance and species richness, whereas negative correlations were found between mean canopy cover and dung beetle abundance and species richness [54]. Although from a completely different biome, these results support our conclusion that in the woodlands, diversity increased with increasing elevation because of decreasing tree cover.

The decline of diversity with elevation in grasslands is likely due to increasingly hostile environmental conditions [53] and cannot be related to the absence of large herbivores at higher elevations in the study area, because during the summer months the cattle are moved from lowland to mountain pastures, where they graze at elevations between 1400 and 2100 m. However, further studies should be performed to reveal how environmental conditions act on species diversity patterns along the gradient.

The strong decrease in diversity with increasing elevation in the grassland assemblages was contrasted by an increase in abundances, especially in the scarabaeids. This increase in abundance was mostly due to the very high abundance values attained by the scarabaeid *Onthophagus fracticornis*, which accounted for more than 90% of the total dung beetles collected in the grasslands at 1700–1850 m and some 80% at 1850–2000 m. Among the aphodiids, *Bodilopsis rufa* was particularly abundant (some 14% of total captured individuals) in the grassland habitat of the highest belt. These two species are eurytopic dung beetles with wide elevational ranges, and the ability of *B. rufa* to dig pedotrophic nests [78] may make this species a superior competitor compared to other aphodiids. The very high abundance of dung beetles observed in the 1700–1850 m can be associated with the distribution of grazing activities in the study area, where, during our sampling, cattle were very numerous and frequent in the pastures located at 1700–1800 m, allowing a larger availability of trophic sources to exist here.

Romero-Alcaraz and Ávila [57] found a positive correlation between dung beetle diversity (expressed as average Shannon diversity per trap) and elevation, which seems to be in contrast with our general trends. In fact, the lack of a significant correlation between richness and elevation and the presence of a positive correlation between the Shannon diversity and elevation found in Romero-Alcaraz and Ávila’s study paralleled our results for the woodlands and can be explained by the fact that their sampling sites were mostly placed in woodlands and shrublands. In general, we found that with increasing elevation, assemblages tend to become less diverse because of the increasing dominance of only a few species, which is consistent with a decrease in equitability found in Alpine dung beetle assemblages along an elevational gradient (1750 to 2760 m) in the Maurienne Valley (France) [45]. Thus, the main effect of elevation was that of decreasing dung beetle diversity because environmental conditions become less favorable; however, the abundance of cattle at a high altitude allows a few species to reach very high values of abundance.

## 5. Conclusions

We found that dung beetle assemblages along a Mediterranean elevational gradient were dominated by scarabaeids, a group of beetles that protect their offspring through subterranean pedotrophic nests. This indicates that warm and dry summer climatic conditions at high elevations favor species that are able to protect their larvae from desiccation. In accordance with our prediction based on the overall preferences of these insects for open habitats, dung beetles were much more abundant and diversified in grasslands than in woodlands. Dung beetle diversity variation with elevation followed opposite patterns in the woodlands and grasslands. In the woodlands, diversity increased with increasing elevation because of decreasing tree cover, which causes the landscape to be more open and hence more favorable to dung beetles. By contrast, in the grasslands, diversity decreased with elevation because of increasingly harsher environmental conditions. These results indicate a trade-off in the beetle response to elevation between the positive effects of the increasing availability of more suitable habitats and the decrease of optimal environmental conditions.

## Figures and Tables

**Table 1:** Number of individuals captured for each species of dung beetle collected along an elevational gradient in central Italy.

Taxonomy	Individuals in Woodlands	Individuals in Grasslands	Total Number of Collected Individuals
Geotrupidae			
*Geotrupes spiniger* Marsham, 1802	17	26	43
*Sericotrupes niger* (Marsham, 1802)	34	107	141
*Trypocopris vernalis* (Linnaeus, 1758)	0	21	21
Aphodiidae			
*Acrossus depressus* (Kugelann, 1792)	1	0	1
*Acrossus rufipes* (Linnaeus, 1758)	40	11	51
*Amidorus obscurus* (Fabricius, 1792)	1	4	5
*Amidorus thermicola* (Sturm, 1800)	0	3	3
*Aphodius coniugatus* (Panzer, 1795)	0	13	13
*Aphodius fimetarius* (Linnaeus, 1758)	0	48	48
*Bodiloides ictericus*(Laicharting, 1781)	0	1	1
*Bodilopsis rufa* (Moll, 1782)	48	673	721
*Chilothorax conspurcatus* (Linnaeus, 1758)	0	1	1
*Chilothorax paykulli* (Bedel, 1907)	0	1	1
*Colobopterus erraticus* (Linnaeus, 1758)	0	35	35
*Coprimorphus scrutator* (Herbst, 1789)	0	1	1
*Esymus pusillus pusillus* (Herbst, 1789)	17	10	27
*Euheptaulacus carinatus* (Germar, 1824)	3	300	303
*Labarrus lividus* (Olivier, 1789)	0	15	15
*Limarus zenkeri* (Germar, 1813)	255	141	396
*Melinopterus consputus* (Creutzer, 1799)	1	3	4
*Melinopterus prodromus* (Brahm, 1790)	0	2	2
*Nimbus contaminatus* (Herbst, 1783)	0	93	93
*Nimbus johnsoni* (Baraud, 1976)	179	55	234
*Nimbus obliteratus* (Panzer, 1823)	21	24	45
*Otophorus haemorrhoidalis* (Linnaeus, 1758)	0	1	1
*Phalacronotus biguttatus* (Germar, 1824)	0	1	1
*Planolinoides borealis* (Gyllenhal, 1827)	1	0	1
*Planolinus fasciatus* (Olivier, 1789)	58	62	120
*Sigorus porcus* (Fabricius, 1792)	3	54	57
*Trichonotulus scrofa* (Fabricius, 1787)	0	1	1
Scarabaeidae			
*Copris umbilicatus* Abeille de Perrin, 1901	0	2	2
*Euoniticellus fulvus* (Goeze, 1777)	0	5	5
*Euonthophagus gibbosus* (Scriba, 1790)	0	4	4
*Onthophagus coenobita* (Herbst, 1783)	3	40	43
*Onthophagus fracticornis* (Preyssler, 1790)	41	7483	7524
*Onthophagus illyricus* (Scopoli, 1763)	0	7	7
*Onthophagus joannae* Goljan, 1953	10	980	990
*Onthophagus lemur* (Fabricius, 1781)	0	32	32
*Onthophagus taurus* (Schreber, 1759)	1	6	7
*Onthophagus vacca* (Linnaeus, 1767)	0	1	1
*Onthophagus verticicornis* (Laicharting, 1781)	47	1065	1112
*Sisyphus schaefferi* (Linnaeus, 1758)	1	210	211
Total	731	11,388	12,324

**Table 2:** Results of generalized linear models for the relationships of diversity (**A**–**C**) and abundance (**D**) of dung beetles with altitude along an elevational gradient in central Italy. Diversity (expressed as species richness, Shannon diversity, and Simpson diversity) was calculated using empirical (**A**), rarefied/interpolated (**B**), and asymptotic (**C**) values. Analyses were conducted for the two habitats (woodlands and grasslands), both separately and merged. SE = standard error, *p* = probability, *R*^2^ = Kullback–Leibler-divergence-based *R*^2^.

	Woodlands				Grasslands				Habitats Merged			
	Estimate	SE	*p*	*R* ^2^	Estimate	SE	*p*	*R* ^2^	Estimate	SE	*p*	*R* ^2^
(A) Empirical diversity values												
Richness	0.0001	0.0005	0.8601	0.0082	0.0002	0.0003	0.5095	0.1219	0.0002	0.0003	0.5030	0.0900
Shannon	0.0040	0.0010	0.0167	0.7965	−0.0090	0.0016	0.0027	0.8591	−0.0079	0.0009	0.0003	0.9438
Simpson	0.0040	0.0002	<0.0001	0.9942	−0.0059	0.0014	0.0070	0.7945	−0.0048	0.0006	0.0004	0.9346
(B) Rarefied/interpolated diversity values												
Richness	0.0002	0.0005	0.7624	0.0491	−0.0012	0.0004	0.0038	0.9321	−0.0005	0.0003	0.0828	0.5295
Shannon	0.0041	0.0007	0.0052	0.8852	−0.0082	0.0015	0.0025	0.8625	−0.0081	0.0009	0.0002	0.9464
Simpson	0.0041	0.0003	0.0002	0.9756	−0.0056	0.0012	0.0063	0.8036	−0.0049	0.0006	0.0004	0.9358
(C) Asymptotic diversity values												
Richness	0.0005	0.0004	0.2103	0.1207	0.0007	0.0002	0.0020	0.8071	0.0004	0.0002	0.0420	0.1734
Shannon	0.0046	0.0011	0.0135	0.8167	−0.0094	0.0017	0.0026	0.8598	−0.0082	0.0009	0.0003	0.9440
Simpson	0.0044	0.0003	0.0002	0.9777	−0.0061	0.0014	0.0074	0.7907	−0.0049	0.0006	0.0004	0.9359
(D) Abundance	−0.0011	0.0001	<0.0001	0.2821	0.0026	<0.0001	<0.0001	0.7431	0.0023	<0.0001	<0.0001	0.7174

**Table 3:** Results of generalized linear models for the relationships between diversity (**A**–**C**) and abundance (**D**) of aphodiid dung beetles and altitude along an elevational gradient in central Italy. Diversity (expressed as species richness, Shannon diversity, and Simpson diversity) was calculated using empirical (**A**), rarefied/interpolated (**B**), and asymptotic (**C**) values. Analyses were conducted for the two habitats (woodlands and grasslands), both separately and merged. SE = standard error, *p* = probability, *R*^2^ = Kullback–Leibler-divergence-based *R*^2^.

	Woodlands				Grasslands				Habitats Merged			
	Estimate	SE	*p*	*R* ^2^	Estimate	SE	*p*	*R* ^2^	Estimate	SE	*p*	*R* ^2^
(A) Empirical diversity values												
Richness	0.0001	0.0006	0.8599	0.0105	0.0003	0.0004	0.3514	0.3269	0.0003	0.0003	0.3590	0.2248
Shannon	0.0028	0.0008	0.0272	0.7432	−0.0043	0.0015	0.0332	0.6294	−0.0006	0.0018	0.7690	0.0189
Simpson	0.0027	0.0005	0.0067	0.8693	−0.0037	0.0012	0.0287	0.6494	−0.0001	0.0012	0.9380	0.0013
(B) Rarefied/interpolated diversity values												
Richness	0.0002	0.0006	0.7041	0.066	−0.0004	0.0004	0.3030	0.5069	<0.0001	0.0003	0.9590	0.0007
Shannon	0.0029	0.0007	0.0147	0.8091	−0.0044	0.0014	0.0250	0.6668	−0.0007	0.0018	0.7043	0.0313
Simpson	0.0028	0.0006	0.0080	0.8573	−0.0037	0.0012	0.0267	0.6584	−0.0001	0.0012	0.9261	0.0019
(C) Asymptotic diversity values												
Richness	0.0004	0.0006	0.4566	0.0972	0.0010	0.0003	0.0022	0.7536	0.0007	0.0003	0.0102	0.3756
Shannon	0.0031	0.0009	0.0214	0.7711	−0.0047	0.0016	0.0286	0.6497	−0.0007	0.0019	0.7347	0.0250
Simpson	0.0029	0.0006	0.0082	0.8560	−0.0040	0.0013	0.0265	0.6594	−0.0001	0.0012	0.9290	0.0018
(D) Abundance	−0.0016	0.0002	<0.0001	0.4444	0.0020	0.0001	<0.0001	0.7263	0.0008	0.0001	<0.0001	0.3090

**Table 4:** Results of generalized linear models for the relationships between diversity (**A**–**C**) and abundance (**D**) of scarabaeid dung beetles and altitude along an elevational gradient in central Italy. Diversity (expressed as species richness, Shannon diversity and Simpson diversity) was calculated using empirical (**A**), rarefied/interpolated (**B**), and asymptotic (**C**) values. Analyses were conducted for the two habitats (woodlands and grasslands), both separately and merged. SE = standard error, *P* = probability, R^2^ = Kullback–Leibler-divergence-based *R*^2^.

	Woodlands				Grasslands				Habitats Merged			
	Estimate	SE	*p*	*R* ^2^	Estimate	SE	*p*	*R* ^2^	Estimate	SE	*p*	*R* ^2^
(A) Empirical diversity values												
Richness	<−0.0001	0.0010	0.9770	0.0036	−0.0002	0.0005	0.6136	0.0492	−0.0002	0.0005	0.6136	0.0492
Shannon	−0.0018	0.0012	0.2242	0.4373	−0.0023	0.0010	0.0807	0.4881	−0.0024	0.0009	0.0510	0.5660
Simpson	−0.0017	0.0011	0.2138	0.4519	−0.0015	0.0009	0.1539	0.3607	−0.0016	0.0008	0.1121	0.4259
(B) Rarefied/interpolated diversity values												
Richness	−0.0011	0.0012	0.3685	0.3867	−0.0008	0.0009	0.3205	0.6141	−0.0005	0.0006	0.4046	0.0716
Shannon	−0.0022	0.0013	0.1841	0.4962	−0.0011	0.0008	0.2327	0.2693	−0.0009	0.0015	0.5661	0.0701
Simpson	−0.0021	0.0012	0.1852	0.4945	−0.0008	0.0007	0.2925	0.2167	−0.0005	0.0011	0.6475	0.0451
(C) Asymptotic diversity values												
Richness	<−0.0001	0.0010	0.9770	0.0036	−0.0002	0.0004	0.6912	0.0145	−0.0001	0.0004	0.8411	0.0036
Shannon	−0.0022	0.0015	0.2436	0.4111	−0.0023	0.0010	0.0721	0.5083	−0.0025	0.0009	0.0465	0.5805
Simpson	−0.0026	0.0016	0.1884	0.4890	−0.0015	0.0009	0.1490	0.3670	−0.0016	0.0008	0.1081	0.4331
(D) Abundance	0.0019	0.0004	<0.0001	0.8419	0.0027	<0.0001	<0.0001	0.6838	0.0027	<0.0001	<0.0001	0.6825

## Data Availability

The data that support the findings of this study are openly available as Supplementary Materials (Table S1).

## Supplementary Materials

The following are available online at https://www.mdpi.com/article/10.3390/insects12090781/s1. Table S1: Number of dung beetles collected in two habitats (woodlands and grasslands along an elevational gradient in central Italy. Figure S1. Diversity curves using Hill numbers for dung beetle assemblages along an elevational gradient in central Italy with confidence intervals, Figure S2: Relationship between dung beetle species richness, exponential Shannon diversity, and Simpson diversity and altitude along an elevational gradient in central Italy. Curves were constructed using values based on rarefaction/extrapolation for the two habitats, both separately and merged, Figure S3: Relationship between dung beetle species richness, exponential Shannon diversity, and Simpson diversity and altitude along an elevational gradient in central Italy. Curves were constructed using asymptotic values for the two habitats, both separately and merged, Figure S4: Diversity curves using Hill numbers for dung beetle assemblages along an elevational gradient in central Italy. Curves were constructed for aphodiids and scarabaeids using empirical values, Figure S5: Diversity curves using Hill numbers for dung beetle assemblages along an elevational gradient in central Italy with confidence intervals. Curves were constructed for aphodiids and scarabaeids using empirical values. Figure S5. Diversity curves using Hill numbers for dung beetle assemblages along an elevational gradient in central Italy constructed for aphodiids and scarabaeids using rarefied/extrapolated values, Figure S6: Diversity curves using Hill numbers for dung beetle assemblages along an elevational gradient in central Italy constructed for aphodiids and scarabaeids using rarefied/extrapolated values with confidence intervals, Figure S7: Diversity curves using Hill numbers for dung beetle assemblages along an elevational gradient in central Italy constructed for aphodiids and scarabaeids using asymptotic values, Figure S8: Diversity curves using Hill numbers for dung beetle assemblages along an elevational gradient in central Italy constructed for aphodiids and scarabaeids using asymptotic values with confidence intervals, Figure S9: Relationship between aphodiid species richness, exponential Shannon diversity, and Simpson diversity and altitude along an elevational gradient in central Italy. Curves were constructed using values based on rarefaction/extrapolation for the two habitats, both separately and merged, Figure S10: Relationship between aphodiid species richness, exponential Shannon diversity, and Simpson diversity and altitude along an elevational gradient in central Italy. Curves were constructed using asymptotic values for the two habitats, both separately and merged, Figure S11: Relationship between scarabaeid species richness, exponential Shannon diversity, and Simpson diversity and altitude along an elevational gradient in central Italy. Curves were constructed using values based on rarefaction/extrapolation for the two habitats, both separately and merged, Figure S12: Relationship between scarabaeid species richness, exponential Shannon diversity, and Simpson diversity and altitude along an elevational gradient in central Italy. Curves were constructed using asymptotic values for the two habitats, both separately and merged.
