# Supplementary material for: Diversity Patterns of Dung Beetles along a Mediterranean Elevational Gradient
Source: Insects. 2021 Aug 31;12(9):781. doi: 10.3390/insects12090781 (PMC8466803; doi:10.3390/insects12090781)
Supplement: Supplementary file 1 [file insects-12-00781-s001.zip › insects-1342676-supplementary.pdf]

Table S1. Number of dung beetles collected in two habitats (woodlands and grasslands along an elevational gradient in central Italy. Dung beetles were sampled in seven altitudinal intervals (belts) of 150 m extent: Belt 1: 950-1100 m, Belt 2: 1100-1250 m, Belt 3: 1250-1400 m, Belt 4: 1400-1550, Belt 5: 1550-1700, Belt 6: 1700-1850, Belt 7: 1850-2000. There are no samples from woodlands in Belt 7 as this habitat was not present at this altitude.

	**Woodlands**						**Grasslands**						
**Belt**	Belt 1	Belt 2	Belt 3	Belt 4	Belt 5	Belt 6	Belt 1	Belt 2	Belt 3	Belt 4	Belt 5	Belt 6	Bel7
**Number of samples**	6	6	5	6	5	6	6	6	6	5	5	5	6
Geotrupidae													
Geotrupes spiniger	0	0	6	3	4	4	9	1	5	0	3	5	3
Sericotrupes niger	13	0	5	12	0	4	23	4	6	18	12	27	17
Trypocopris vernalis	0	0	0	0	0	0	0	0	0	0	2	1	18
Aphodiidae													
Acrossus depressus	0	0	0	0	0	1	0	0	0	0	0	0	0
Acrossus rufipes	3	1	0	8	7	21	3	0	6	0	2	0	0
Amidorus obscurus	0	1	0	0	0	0	0	0	0	0	0	0	4
Amidorus thermicola	0	0	0	0	0	0	0	1	0	0	2	0	0
Aphodius coniugatus	0	0	0	0	0	0	1	4	0	0	0	5	3
Aphodius fimetarius	0	0	0	0	0	0	8	3	0	0	6	14	17
Bodiloides ictericus	0	0	0	0	0	0	0	0	0	0	1	0	0
Bodilopsis rufa	7	0	8	7	13	13	27	6	11	29	78	189	333
Chilothorax conspurcatus	0	0	0	0	0	0	0	0	0	0	0	0	1
Chilothorax paykulli	0	0	0	0	0	0	0	0	0	0	0	1	0
Colobopterus erraticus	0	0	0	0	0	0	0	1	0	2	17	5	10
Coprimorphus scrutator	0	0	0	0	0	0	0	0	0	0	0	1	0
Esymus pusillus	5	1	0	5	3	3	4	0	0	2	4	0	0
Euheptaulacus carinatus	0	0	0	2	0	1	0	0	0	1	102	83	114
Labarrus lividus	0	0	0	0	0	0	0	3	1	3	0	4	4
Limarus zenkeri	35	49	50	73	13	35	21	23	76	18	2	0	1
Melinopterus consputus	1	0	0	0	0	0	0	0	1	0	0	1	1
Melinopterus prodromus	0	0	0	0	0	0	2	0	0	0	0	0	0
Nimbus contaminatus	0	0	0	0	0	0	2	0	0	0	64	16	11
Nimbus johnsoni	170	5	1	1	1	1	44	4	2	0	3	1	1
Nimbus obliteratus	7	4	0	8	0	2	3	2	4	1	6	6	2
Otophorus haemorrhoidalis	0	0	0	0	0	0	0	0	0	0	0	1	0
Phalacronotus biguttatus	0	0	0	0	0	0	0	0	0	1	0	0	0
Planolinoides borealis	0	0	0	1	0	0	0	0	0	0	0	0	0
Planolinus fasciatus	16	6	16	13	0	7	20	10	32	0	0	0	0
Sigorus porcus	0	0	1	2	0	0	16	17	1	6	9	4	1
Trichonotulus scrofa	0	0	0	0	0	0	0	0	0	0	1	0	0
Scarabaeidae													
Copris umbilicatus	0	0	0	0	0	0	0	0	0	1	1	0	0
Euoniticellus fulvus	0	0	0	0	0	0	0	0	0	1	2	2	0
Euonthophagus gibbosus	0	0	0	0	0	0	0	0	0	0	1	3	0
Onthophagus coenobita	3	0	0	0	0	0	36	3	0	1	0	0	0
Onthophagus fracticornis	0	2	1	1	0	37	88	6	193	137	1564	3587	1908

Onthophagus illyricus	0	0	0	0	0	0	2	0	0	0	5	0	0
Onthophagus joannae	0	1	7	1	0	1	5	29	20	576	312	24	14
Onthophagus lemur	0	0	0	0	0	0	1	11	0	16	2	1	1
Onthophagus taurus	0	0	1	0	0	0	0	2	3	1	0	0	0
Onthophagus vacca	0	0	0	0	0	0	0	0	0	0	1	0	0
Onthophagus verticicornis	3	16	4	19	4	1	8	141	7	382	525	2	0
Sisyphus schaefferi	1	0	0	0	0	0	1	78	4	112	14	1	0

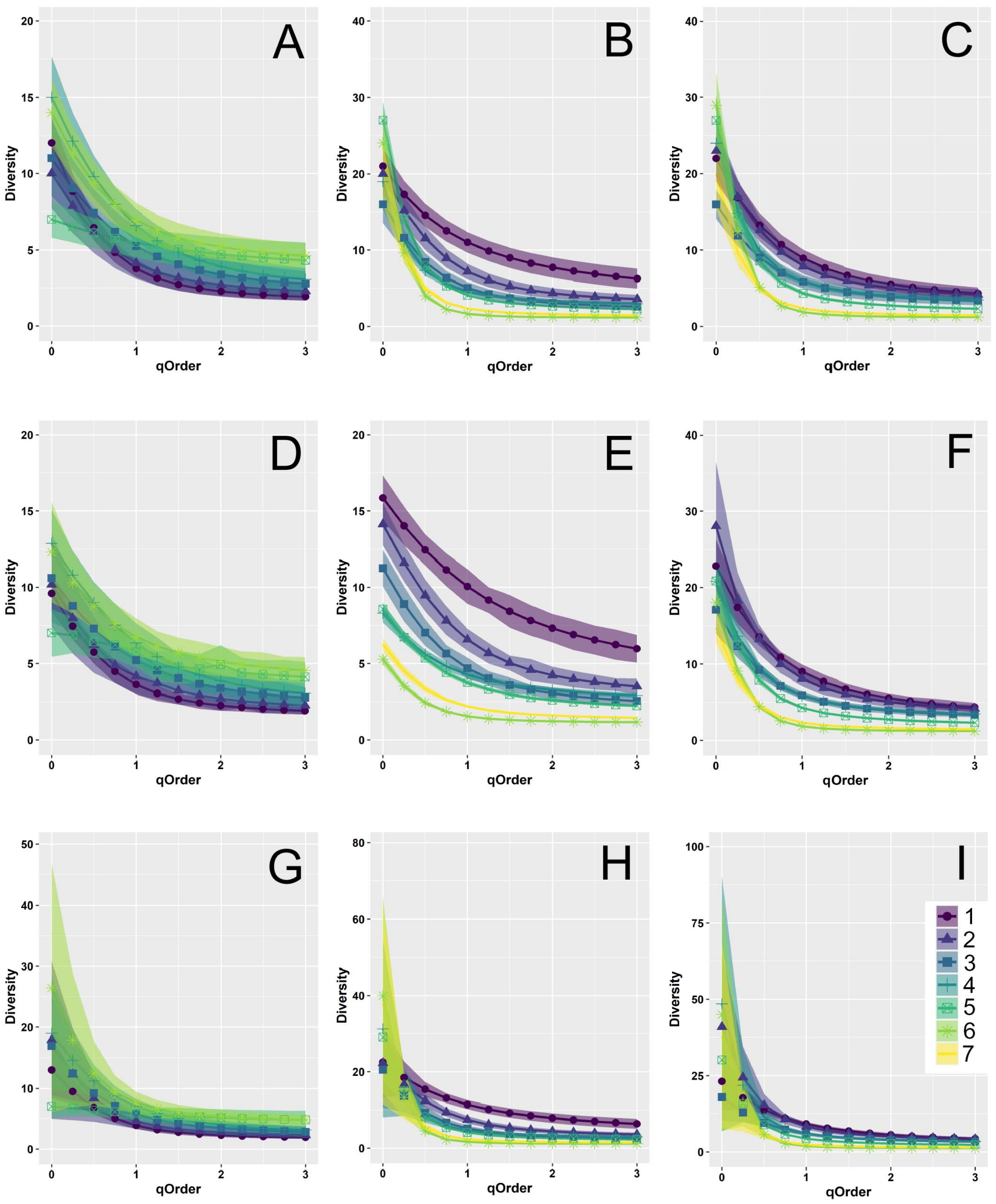

Figure S1. Diversity curves using Hill numbers for dung beetle assemblages along an elevational gradient in central Italy with confidence intervals. Curves were constructed using empirical (**A**, **B**, **C**), rarefied/extrapolated (**D**, **E**, **F**) and asymptotic (**G**, **H**, **I**) values for the two habitats (woodlands and grasslands) separately (woodlands: **A**, **D**, **G**; grasslands: **B**, **E**, **H**) and merged (**C**, **F**, **I**). Dung beetles were sampled in seven altitudinal intervals (belts) of 150 m extent: Belt 1: 950-1100 m, Belt 2: 1100-1250 m, Belt 3: 1250-1400 m, Belt 4: 1400-1550, Belt 5: 1550-1700, Belt 6: 1700-1850, Belt 7: 1850-2000. 95% confidence intervals are shown as shaded areas.

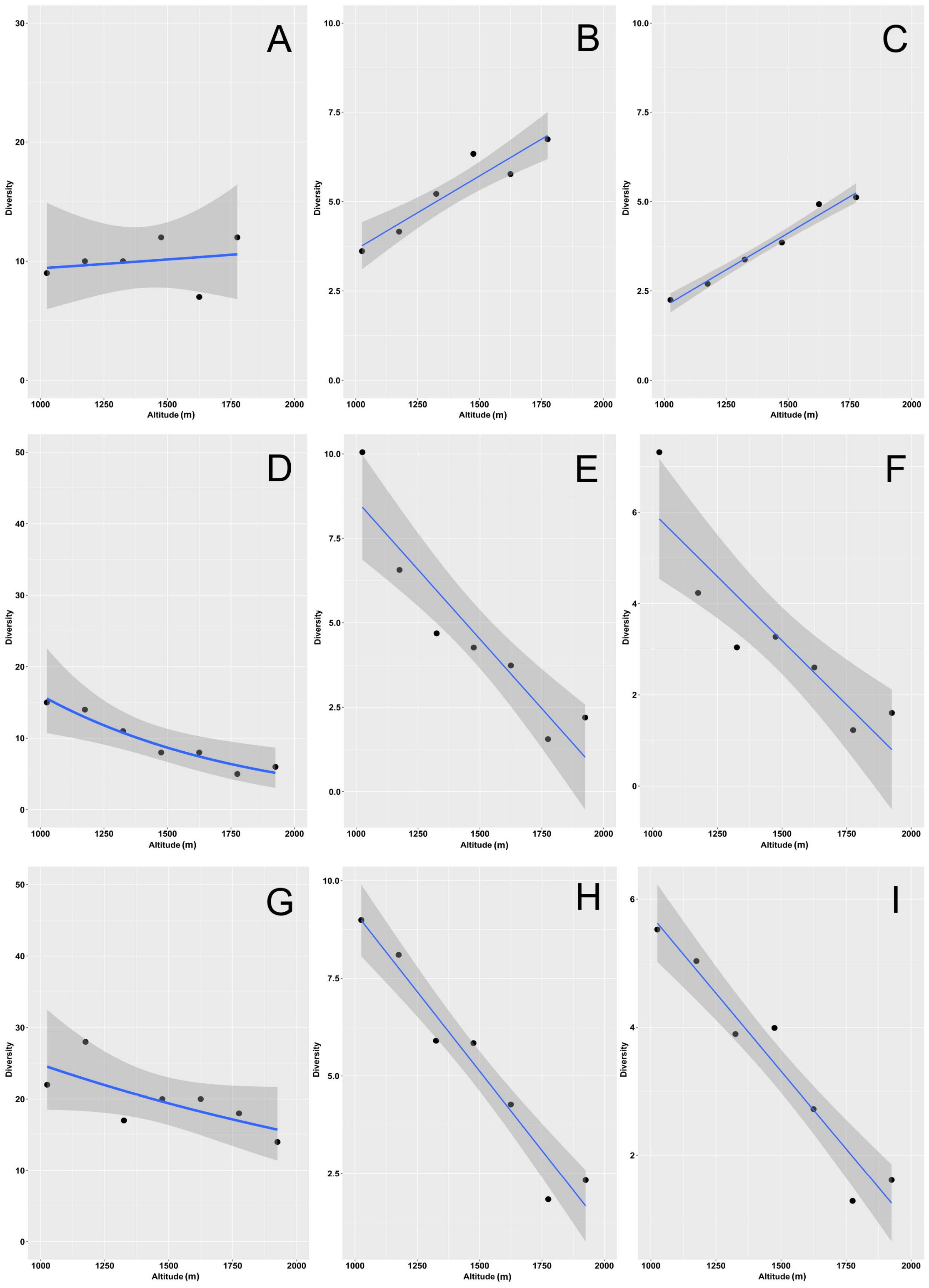

Figure S2. Relationship between dung beetle species richness (**A**, **D**, **G**), exponential Shannon diversity (**B**, **E**, **H**) and Simpson diversity (**C**, **F**, **I**) and altitude along an elevational gradient in central Italy. Curves were constructed using values based on rarefaction/extrapolation for the two habitats (woodlands and grasslands) separately (woodlands: **A**, **B**, **C**; grasslands: **D**, **E**, **F**) and merged (**G**, **H**, **I**). General linear models were used for fitting. Dashed areas are 95% confidence intervals.

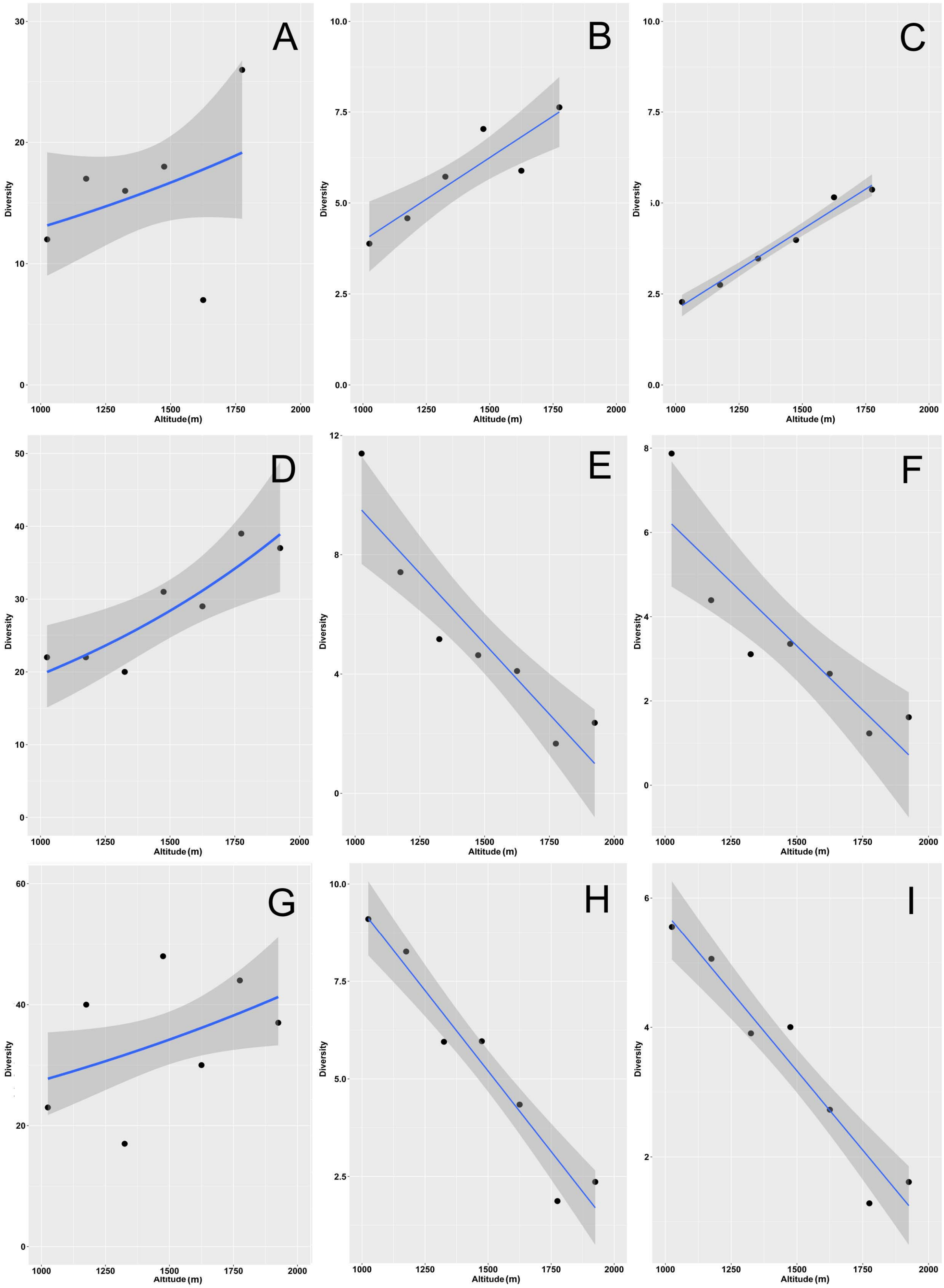

Figure S3. Relationship between dung beetle species richness (**A**, **D**, **G**), exponential Shannon diversity (**B**, **E**, **H**) and Simpson diversity (**C**, **F**, **I**) and altitude along an elevational gradient in central Italy. Curves were constructed using asymptotic values for the two habitats (woodlands and grasslands) separately (woodlands: **A**, **B**, **C**; grasslands: **D**, **E**, **F**) and merged (**G**, **H**, **I**). General linear models were used for fitting. Dashed areas are 95% confidence intervals.

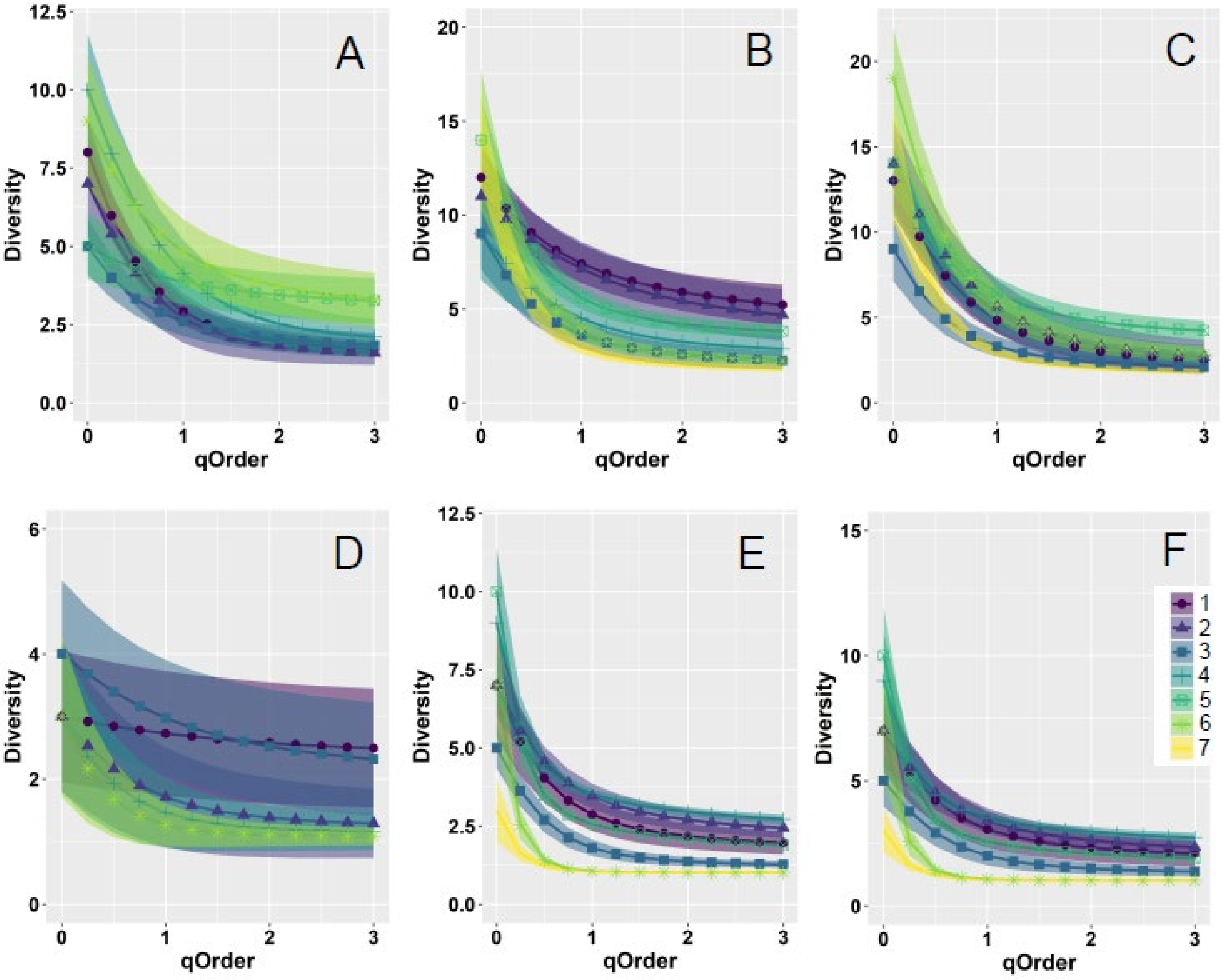

Figure S4. Diversity curves using Hill numbers for dung beetle assemblages along an elevational gradient in central Italy. Curves were constructed for aphodiids (**A-C**) and scarabaeids (**D-F**) using empirical values for the two habitats (woodlands and grasslands) separately (woodlands: **A**, **D**; grasslands: **B**, **E**) and merged (**C**, **F**). Dung beetles were sampled in seven altitudinal intervals (belts) of 150 m extent: Belt 1: 950-1100 m, Belt 2: 1100-1250 m, Belt 3: 1250-1400 m, Belt 4: 1400-1550, Belt 5: 1550-1700, Belt 6: 1700-1850, Belt 7: 1850-2000. 95% confidence intervals are shown as shaded areas.

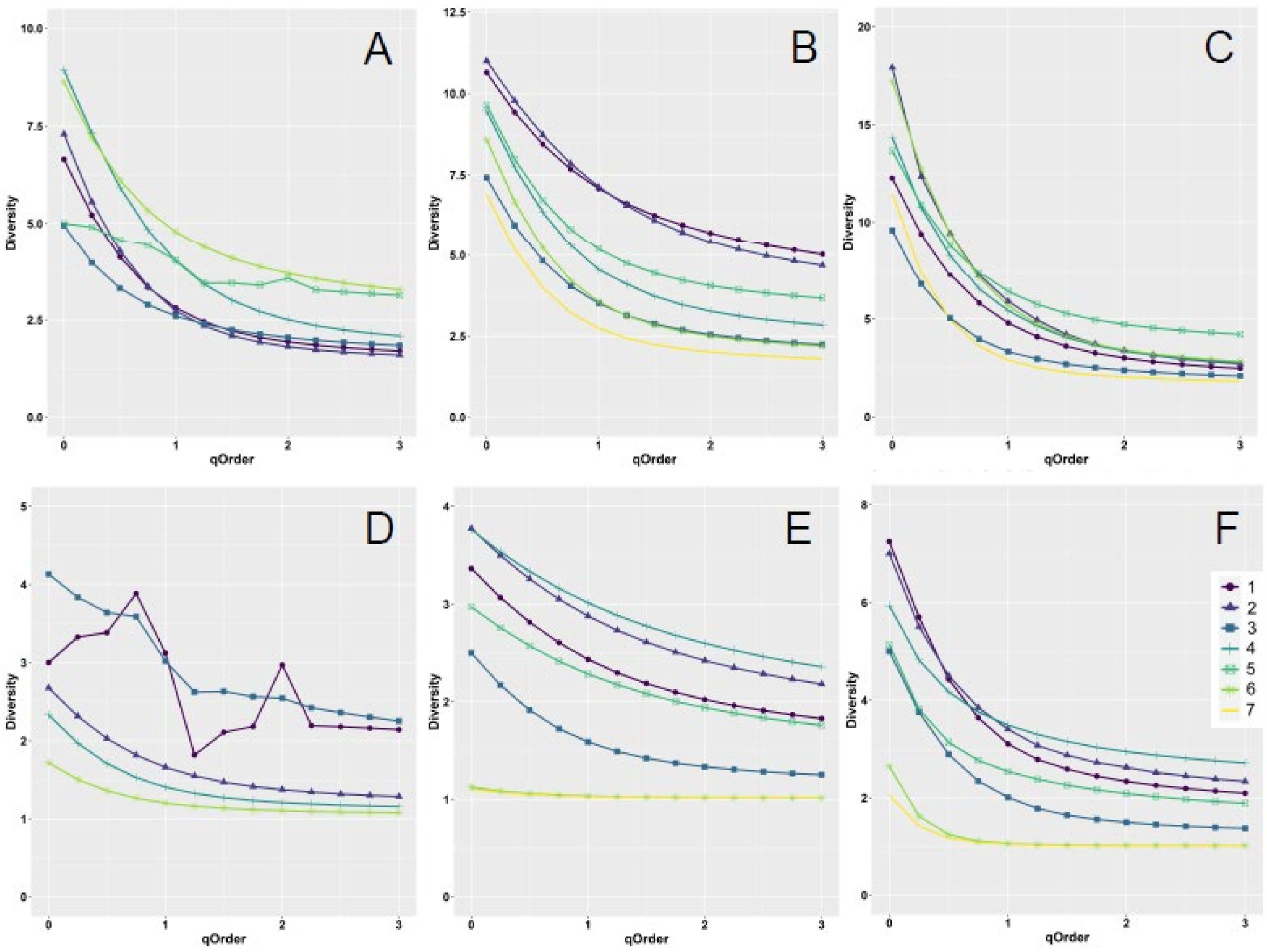

Figure S5. Diversity curves using Hill numbers for dung beetle assemblages along an elevational gradient in central Italy. Curves were constructed for aphodiids (**A-C**) and scarabaeids (**D-F**) using rarefied/extrapolated values for the two habitats (woodlands and grasslands) separately (woodlands: **A**, **D**; grasslands: **B**, **E**) and merged (**C**, **F**). Dung beetles were sampled in seven altitudinal intervals (belts) of 150 m extent: Belt 1: 950-1100 m, Belt 2: 1100-1250 m, Belt 3: 1250-1400 m, Belt 4: 1400-1550, Belt 5: 1550-1700, Belt 6: 1700-1850, Belt 7: 1850-2000. For ease of reading, 95% confidence intervals are not shown here, but they are reported in Figure S6.

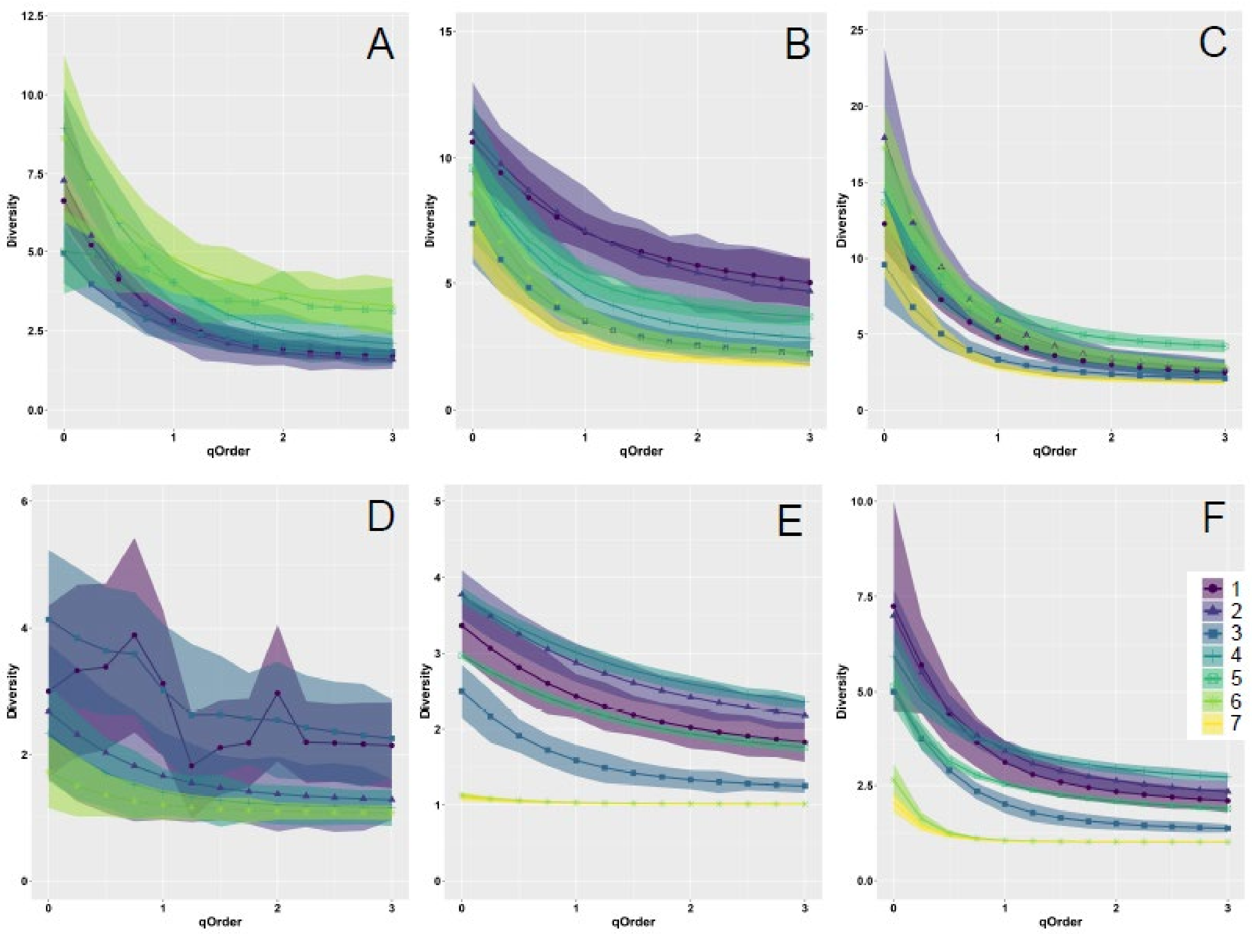

Figure S6. Diversity curves using Hill numbers for dung beetle assemblages along an elevational gradient in central Italy. Curves were constructed for aphodiids (**A-C**) and scarabaeids (**D-F**) using rarefied/extrapolated values for the two habitats (woodlands and grasslands) separately (woodlands: **A**, **D**; grasslands: **B**, **E**) and merged (**C**, **F**). Dung beetles were sampled in seven altitudinal intervals (belts) of 150 m extent: Belt 1: 950-1100 m, Belt 2: 1100-1250 m, Belt 3: 1250-1400 m, Belt 4: 1400-1550, Belt 5: 1550-1700, Belt 6: 1700-1850, Belt 7: 1850-2000. 95% confidence intervals are shown as shaded areas.

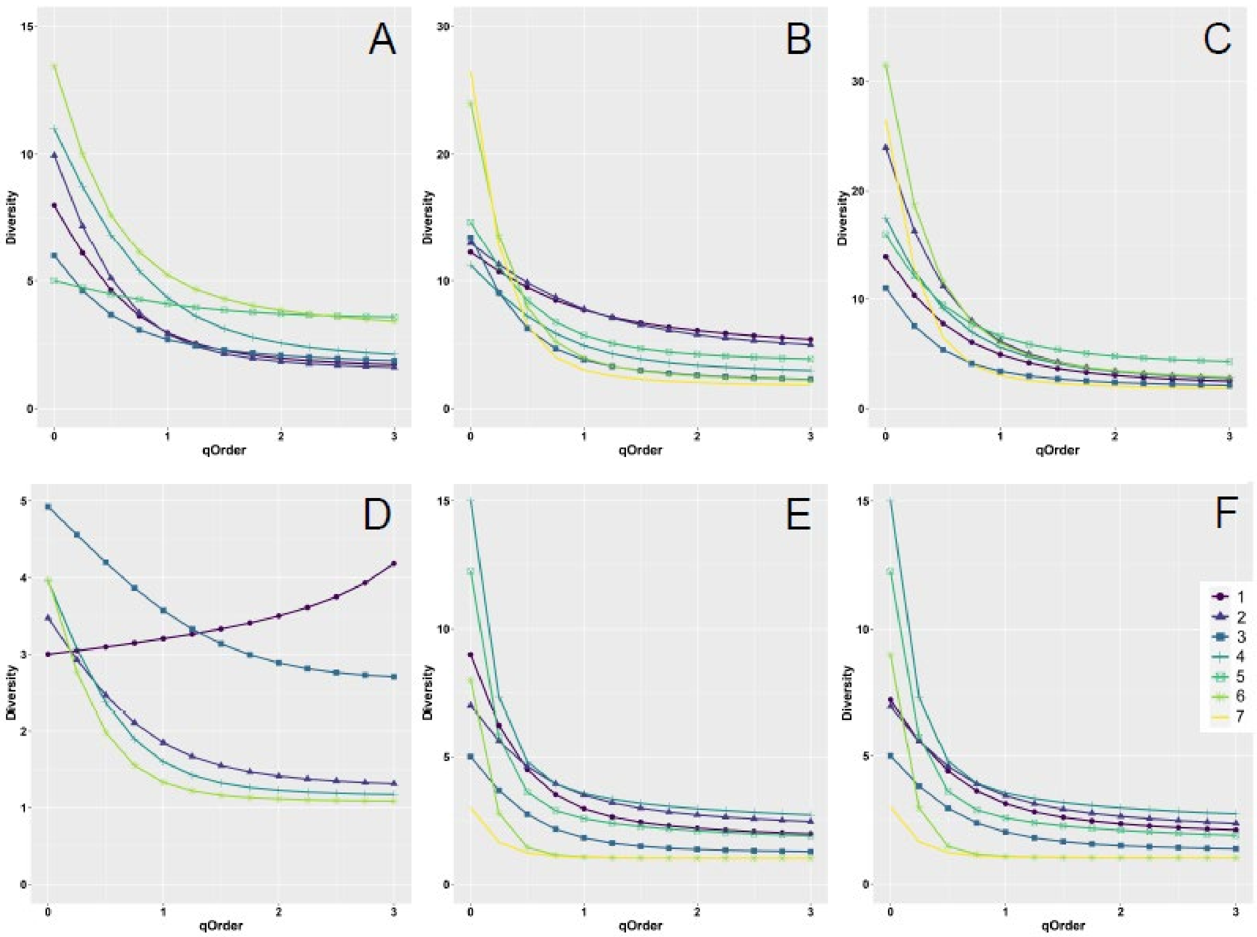

Figure S7. Diversity curves using Hill numbers for dung beetle assemblages along an elevational gradient in central Italy. Curves were constructed for aphodiids (**A-C**) and scarabaeids (**D-F**) using asymptotic values for the two habitats (woodlands and grasslands) separately (woodlands: **A**, **D**; grasslands: **B**, **E**) and merged (**C**, **F**). Dung beetles were sampled in seven altitudinal intervals (belts) of 150 m extent: Belt 1: 950-1100 m, Belt 2: 1100-1250 m, Belt 3: 1250-1400 m, Belt 4: 1400-1550, Belt 5: 1550-1700, Belt 6: 1700-1850, Belt 7: 1850-2000. For ease of reading, 95% confidence intervals are not shown here, but they are reported in Figure S8.

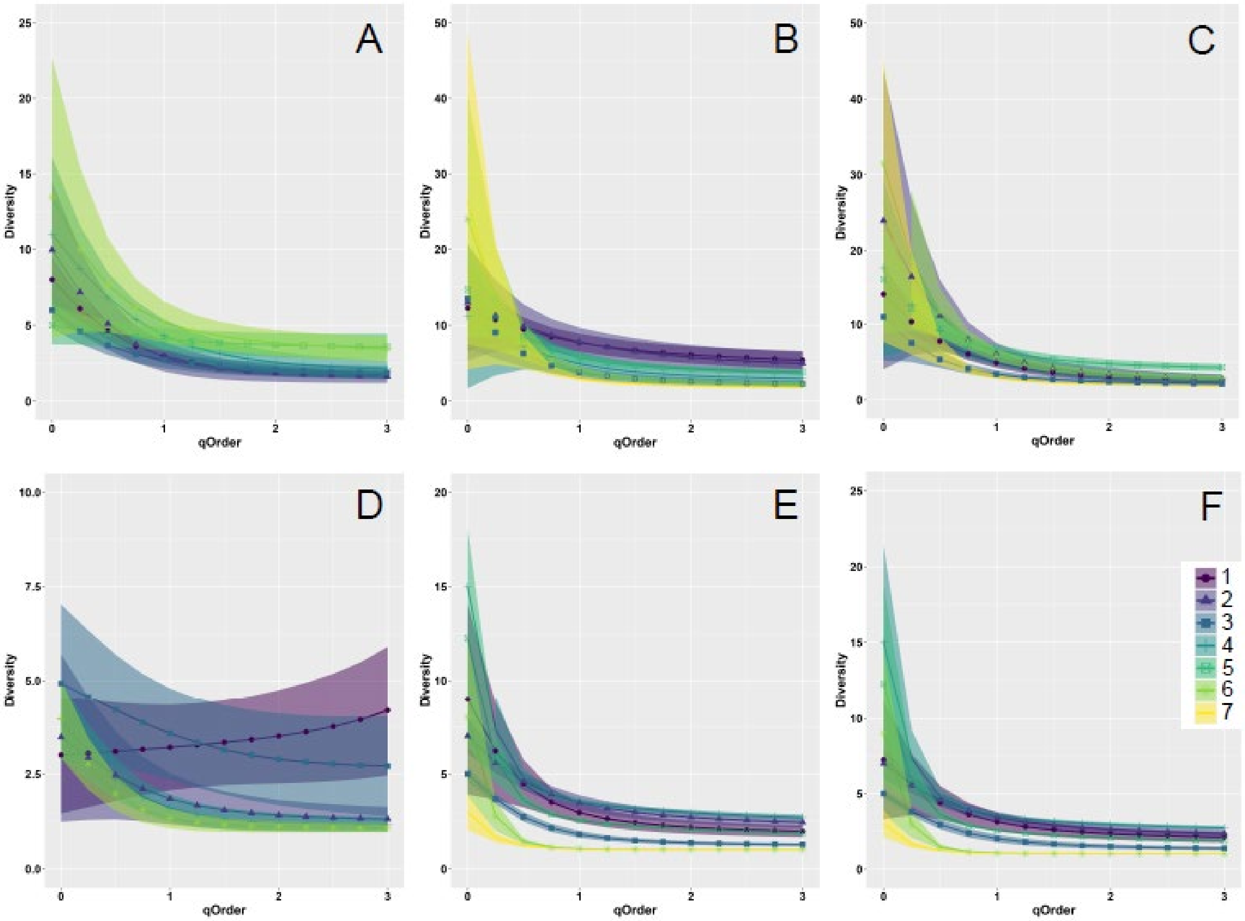

Figure S8. Diversity curves using Hill numbers for dung beetle assemblages along an elevational gradient in central Italy. Curves were constructed for aphodiids (**A-C**) and scarabaeids (**D-F**) using asymptotic values for the two habitats (woodlands and grasslands) separately (woodlands: **A**, **D**; grasslands: **B**, **E**) and merged (**C**, **F**). Dung beetles were sampled in seven altitudinal intervals (belts) of 150 m extent: Belt 1: 950-1100 m, Belt 2: 1100-1250 m, Belt 3: 1250-1400 m, Belt 4: 1400-1550, Belt 5: 1550-1700, Belt 6: 1700-1850, Belt 7: 1850-2000. 95% confidence intervals are shown as shaded areas.

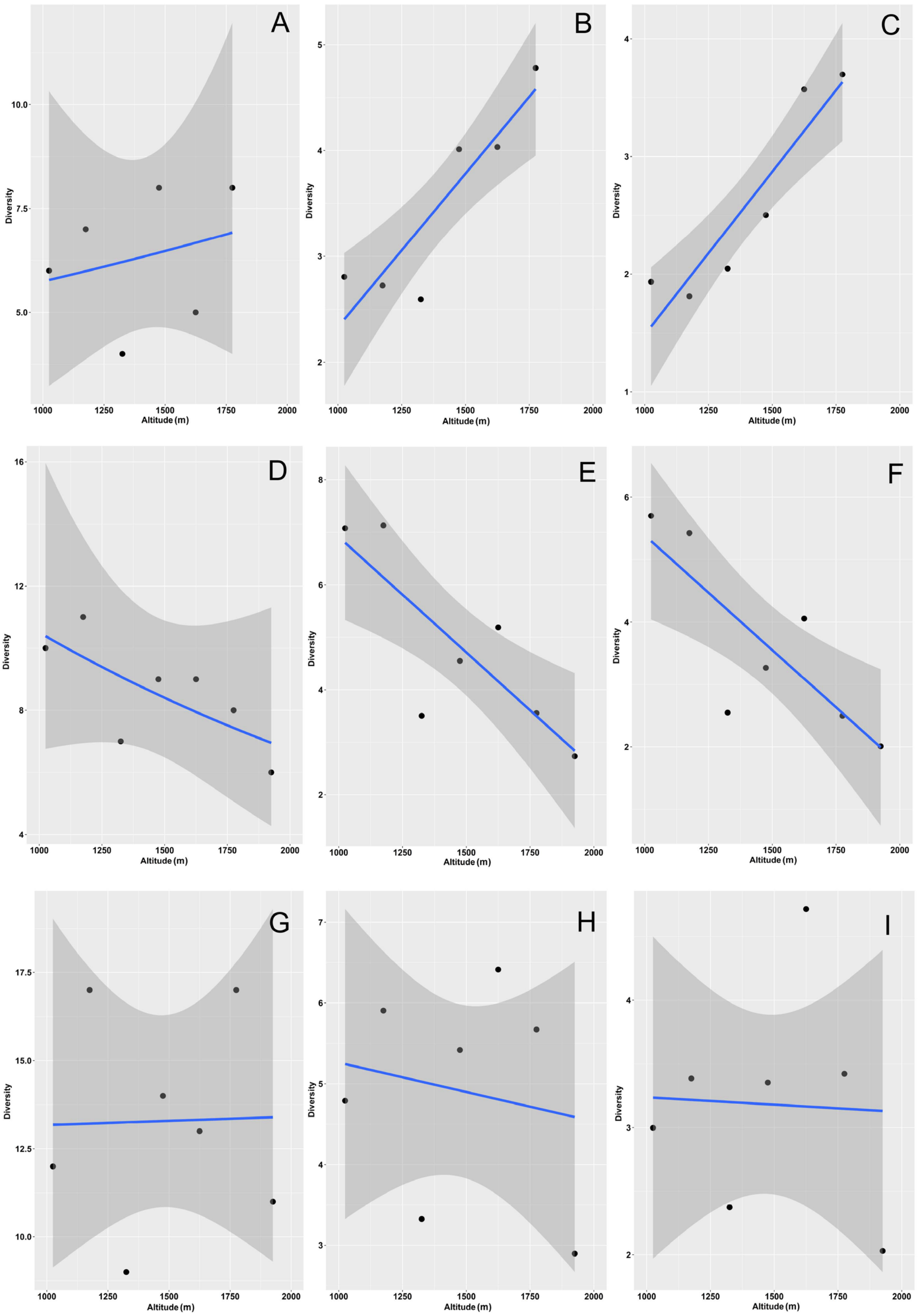

Figure S9. Relationship between aphodiid species richness ((**A**, **D**, **G**), exponential Shannon diversity (**B**, **E**, **H**) and Simpson diversity (**C**, **F**, **I**) and altitude along an elevational gradient in central Italy. Curves were constructed using values based rarefaction/extrapolation for the two habitats (woodlands and grasslands) separately (woodlands: **A**, **B**, **C**; grasslands: **D**, **E**, **F**) and merged (**G**, **H**, **I**). General linear models were used for fitting. Dashed areas are 95% confidence intervals.

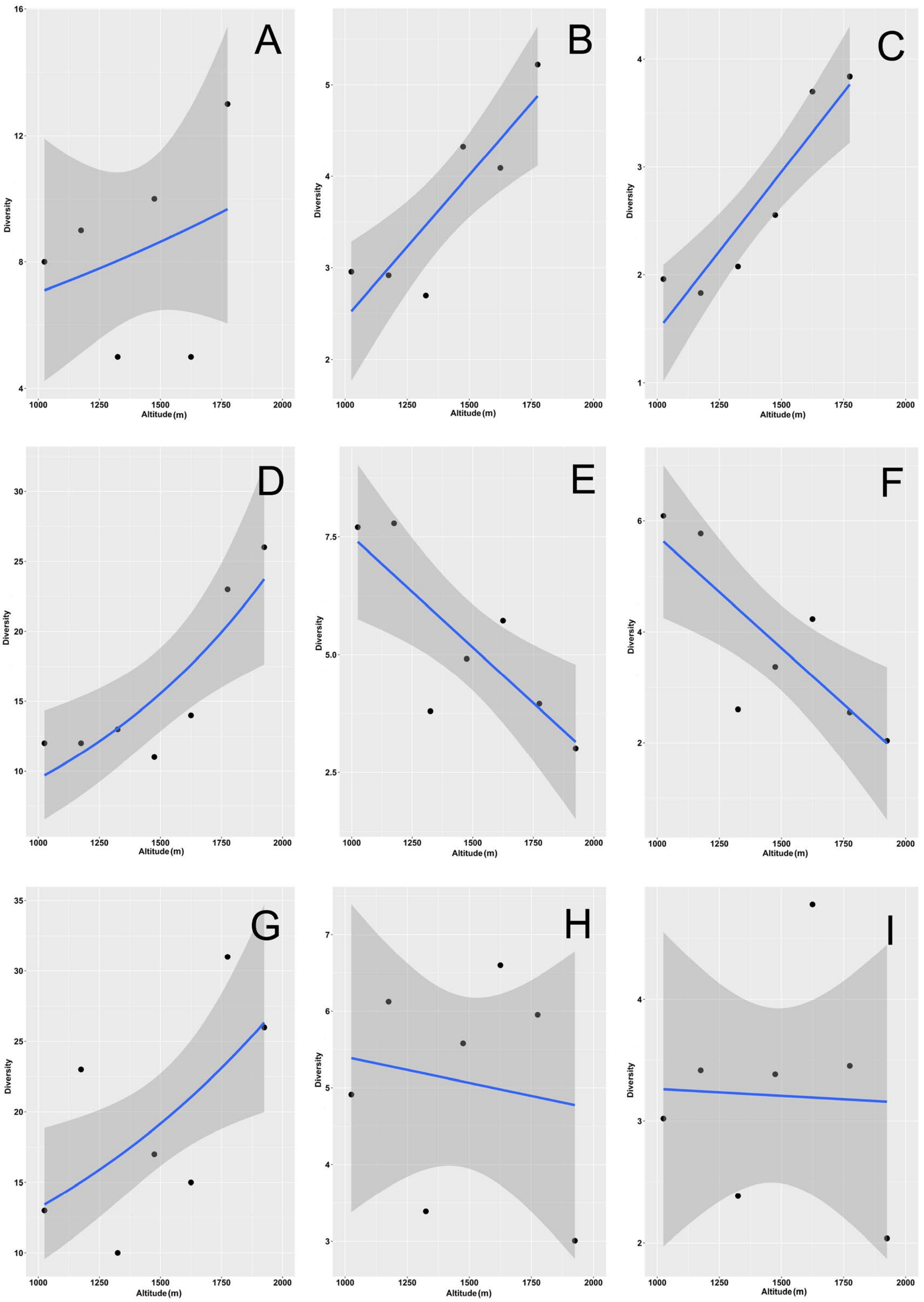

Figure S10. Relationship between aphodiid species richness (A **A**, **D**, **G**), exponential Shannon diversity (**B**, **E**, **H**) and Simpson diversity (**C**, **F**, **I**)) and altitude along an elevational gradient in central Italy. Curves were constructed using asymptotic values for the two habitats (woodlands and grasslands) separately (woodlands: **A**, **B**, **C**; grasslands: **D**, **E**, **F**) and merged (**G**, **H**, **I**). General linear models were used for fitting. Dashed areas are 95% confidence intervals.

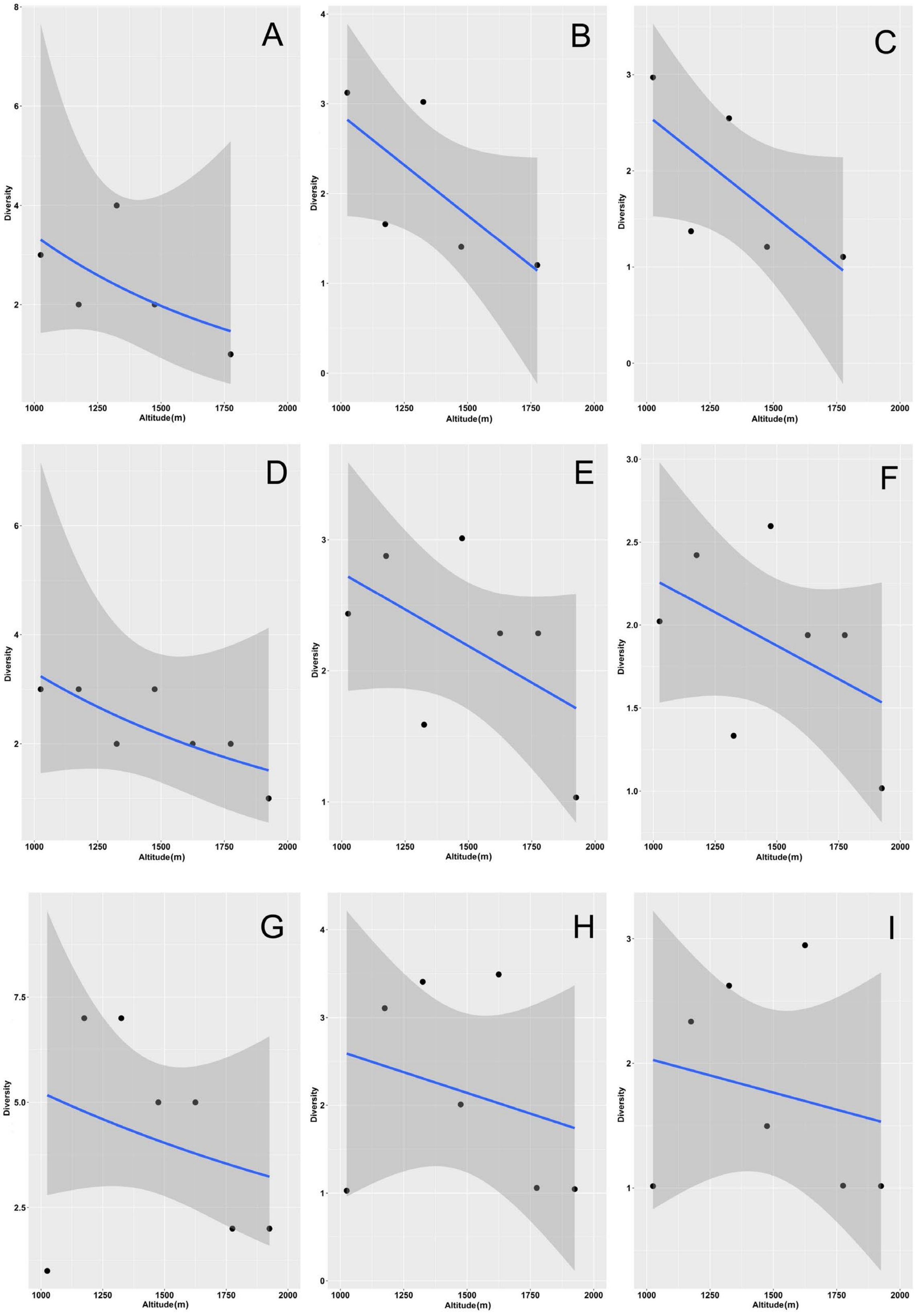

Figure S11. Relationship between scarabaeid species richness r(**A**, **D**, **G**), exponential Shannon diversity (**B**, **E**, **H**) and Simpson diversity (**C**, **F**, **I**) and altitude along an elevational gradient in central Italy. Curves were constructed using values based on rarefaction/extrapolation for the two habitats (woodlands and grasslands) separately (woodlands: **A**, **B**, **C**; grasslands: **D**, **E**, **F**) and merged (**G**, **H**, **I**). General linear models were used for fitting. Dashed areas are 95% confidence intervals.

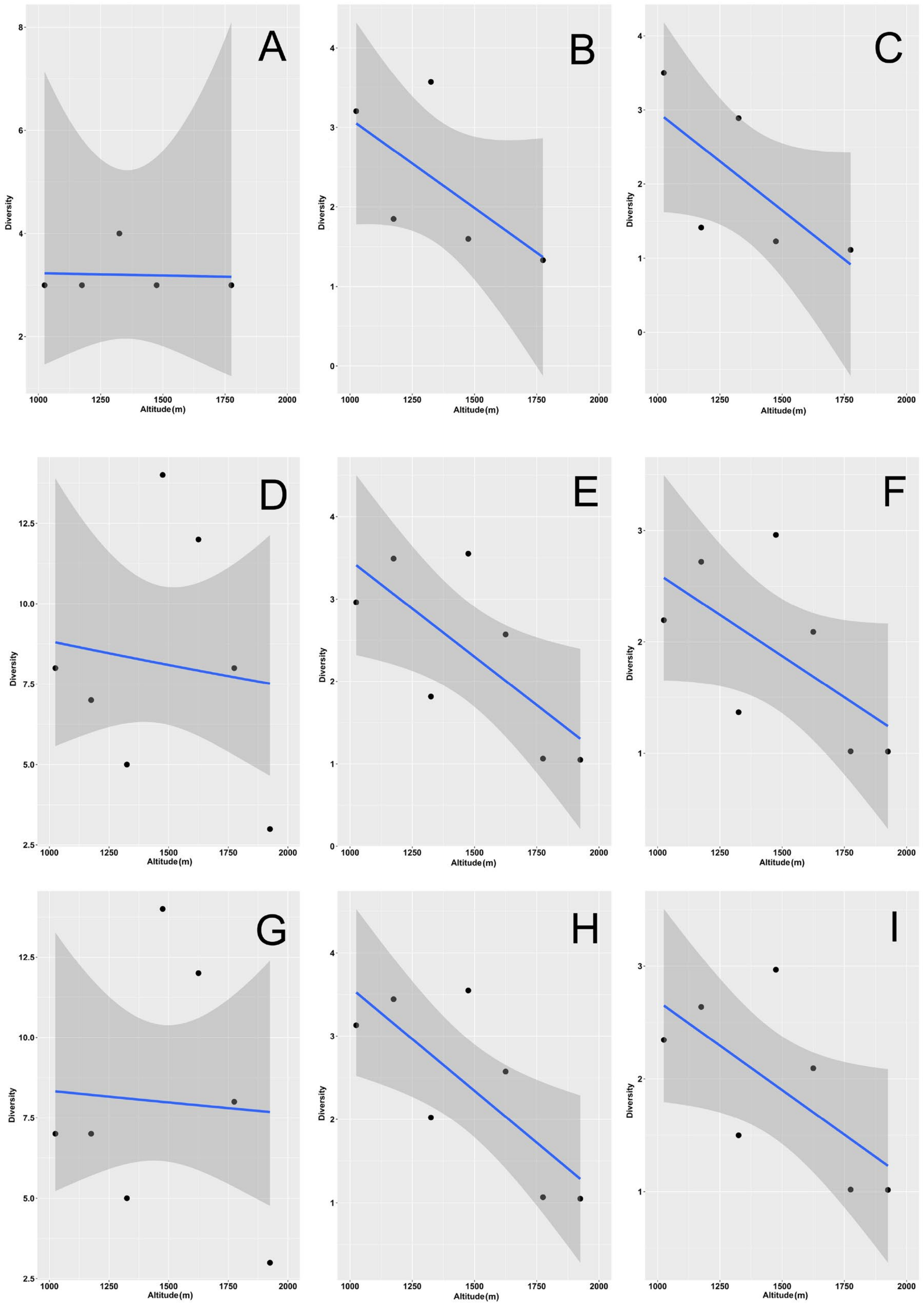

Figure S12. Relationship between scarabaeid-species richness (**A**, **D**, **G**), exponential Shannon diversity (**B**, **E**, **H**) and Simpson diversity (**C**, **F**, **I**) and altitude along an elevational gradient in central Italy. Curves were constructed using asymptotic values for the two habitats (woodlands and grasslands) separately (woodlands: **A**, **B**, **C**; grasslands: **D**, **E**, **F**) and merged (**G**, **H**, **I**). General linear models were used for fitting. Dashed areas are 95% confidence intervals.
